# Nitric Oxide Mediated Transcriptome Profiling Reveals Activation of Multiple Regulatory Pathways in *Arabidopsis thaliana*

**DOI:** 10.3389/fpls.2016.00975

**Published:** 2016-06-29

**Authors:** Adil Hussain, Bong-Gyu Mun, Qari M. Imran, Sang-Uk Lee, Teferi A. Adamu, Muhammad Shahid, Kyung-Min Kim, Byung-Wook Yun

**Affiliations:** ^1^Department of Agriculture, Abdul Wali Khan University MardanMardan, Pakistan; ^2^Laboratory of Plant Functional Genomics, School of Applied Biosciences, Kyungpook National UniversityDaegu, South Korea; ^3^Laboratory of Plant Molecular Breeding, School of Applied Biosciences, Kyungpook National UniversityDaegu, South Korea

**Keywords:** *Arabidopsis thaliana* transcriptomic analysis, nitrosative stress, RNA-Seq profiling, RNS signaling

## Abstract

Imbalance between the accumulation and removal of nitric oxide and its derivatives is a challenge faced by all plants at the cellular level, and is especially important under stress conditions. Exposure of plants to various biotic and abiotic stresses causes rapid changes in cellular redox tone potentiated by the rise in reactive nitrogen species that serve as signaling molecules in mediating defensive responses. To understand mechanisms mediated by these signaling molecules, we performed a large-scale analysis of the Arabidopsis transcriptome induced by nitrosative stress. We generated an average of 84 and 91 million reads from three replicates each of control and 1 mM S-nitrosocysteine (CysNO)-infiltrated Arabidopsis leaf samples, respectively. After alignment, more than 95% of all reads successfully mapped to the reference and 32,535 genes and 55,682 transcripts were obtained. CysNO infiltration caused differential expression of 6436 genes (3448 up-regulated and 2988 down-regulated) and 6214 transcripts (3335 up-regulated and 2879 down-regulated) 6 h post-infiltration. These differentially expressed genes were found to be involved in key physiological processes, including plant defense against various biotic and abiotic stresses, hormone signaling, and other developmental processes. After quantile normalization of the FPKM values followed by student's *T*-test (*P* < 0.05) we identified 1165 DEGs (463 up-regulated and 702 down-regulated) with at least 2-folds change in expression after CysNO treatment. Expression patterns of selected genes involved in various biological pathways were verified using quantitative real-time PCR. This study provides comprehensive information about plant responses to nitrosative stress at transcript level and would prove helpful in understanding and incorporating mechanisms associated with nitrosative stress responses in plants.

## Introduction

Nitric oxide (NO), a highly reactive free radical, is an essential cellular regulatory molecule involved in a plethora of physiological processes in both animal and plant cells. As such, NO has been the center of attention in many fields of research. The discovery of its function as a cardiovascular signal led to the 1998 Nobel Prize for Physiology and Medicine, and was named the “Molecule of the Year” in 1992 by *Science* (Culotta and Koshland, 1992). Since its discovery in mammalian systems in 1987 (Moncada and Palmer, 1993), substantial research regarding NO has been conducted and published in leading research journals, such as *Nature* (Delledonne et al., 1998; Barouch et al., 2002; Nott et al., 2008; Yun et al., 2011) and *Science* (Guo et al., 2003; Matsumoto et al., 2003; He et al., 2004; Tada et al., 2008; Gusarov et al., 2009).

In plants, NO has a well-established role in key physiological processes relating to plant development and immunity (Feechan et al., 2005; Kwon et al., 2012). NO, being highly reactive and therefore toxic to plants, needs to be converted into a non-toxic, mobile, and easily available form. S-nitrosoglutathione (GSNO) is an abundant and bio-available source of NO in the cell. NO can covalently bind to proteins at their solvent-exposed Cys residues to form S-nitrosothiols (SNOs), a phenomenon called S-nitrosylation. Various NO-derivatives, collectively termed reactive nitrogen species (RNS), and especially GSNO and other SNOs, serve as NO donors or carriers in cells and release NO when and where it is required. The global SNO level in plants is controlled by GSNO reductase through the process of de-nitrosylation (Malik et al., 2011).

NO was first found to regulate plant defenses against bacterial pathogens (Delledonne et al., 1998; Durner et al., 1998). Subsequent research involving NO has been shown to mediate many different physiological functions including seed germination, cell expansion, root and flower development, stomatal movement, resistance against biotic, and abiotic stresses (Wilson et al., 2008), and many other functions. While new roles for NO in plants are being elucidated, the exact details of its involvement in complex pathways are still unknown. Though the importance of NO in plant biology is now fully recognized, its main source of production remains largely elusive and the hunt continues for a standard plant nitric oxide synthase (NOS). The only NOS from the plant kingdom to be identified was discovered in the single-cell alga *Ostreococcus tauri*. This enzyme has properties similar to those of mammalian NOS, including the K_m_ of NADPH oxidation and the conversion of Arginine (*Arg*) to citrulline, and exhibits NOS activity *in vitro* (Foresi et al., 2010). It has also been shown that NO is generated in plants through a chemical reaction that is similar to that used in animal systems, i.e., the conversion of *Arg* to citrulline and NO. This oxidative mechanism has also been shown to be significantly reduced in the presence of mammalian NOS inhibitors (Delledonne et al., 2001; Corpas et al., 2006), thus suggesting the presence of NOS-like activity in plants. However, a standard NOS has not yet been characterized in higher plants. The reduction of nitrate by nitrate reductase (NR) is also an important mechanism in the production of NO in plants (Hoff et al., 1992; Yamasaki, 2000; Okada et al., 2004; Ferrarini et al., 2008).

The process of S-nitrosylation is perhaps the most studied regulatory function mediated by NO in plants. Gene products involved in key physiological processes that have been shown to be S-nitrosylated include the Arabidopsis Salicylic Acid Binding Protein-3 (Wang et al., 2009), Non-Expressor of Pathogenesis Related Genes-1 (Tada et al., 2008), and the auxin receptor Transport Inhibitor Response-1 (Terrile et al., 2012). A more recent study identified the largest collection of endogenously S-nitrosylated proteins in any organism to date (Hu et al., 2015) by using a site-specific identification technique in the Arabidopsis *gsnor1-3* knockout line, which accumulates substantial levels of SNOs, and has a disrupted plant defense system (Feechan et al., 2005). The list of S-nitrosylated proteins identified includes 1195 peptides and 926 proteins involved in a large array of physiological processes.

Regulation of protein function by NO through post-translational modifications such as S-nitrosylation and tyrosine nitration has been well-established. However, there is a substantial lack of information about the “inductive or repressive” effects of NO on gene expression (Grün et al., 2006). Biotic and abiotic stresses result in the rise of cellular RNS causing rapid changes in the transcription of various genes. In a study involving a whole-genome based approach, as many as 71 genes exhibited differential expression in response to sodium nitroprusside (SNP; an NO donor) infiltration (Polverari et al., 2003). These genes were found to be involved in plant defense, signal transduction, reactive oxygen species (ROS) production and turnover, photosynthesis, and cell transport. However, the expression of most of these genes is also affected by other biotic and abiotic stresses. Parani et al. (2004), using microarray analysis of Arabidopsis roots treated with 0.1 and 1 mM SNP, reported differential expression of 422 genes (342 up-regulated and 80 down-regulated) involved in plant defense, oxidative stress tolerance, signal transduction, and transcription factors involved in ethylene and abscisic acid (ABA)-dependent pathways. Similarly, many other studies have described transcriptional regulation of various genes by NO (Bolwell, 1999; Kawakita et al., 2004, 2010; Wang et al., 2006). Bioinformatics tools have now made it possible to identify NO-responsive elements in genes and gene promoters.

Transcriptome analysis of Arabidopsis leaves and roots treated with 1 mM GSNO for 3 h revealed differential expression of 3263 genes involved in many physiological processes, including biotic and abiotic stress tolerance (Begara-Morales et al., 2014). Similarly, other Arabidopsis transcriptome analyses have been performed to measure responses to various stimuli such as oxidative stress (Desikan et al., 2001), nitrogen limitation (Peng et al., 2007), phosphate starvation (Woo et al., 2012), nanoparticles (García-Sánchez et al., 2015), cold stress (Zou and Yu, 2010), salt stress and ABA treatment (Matsui et al., 2008), auxin (Paponov et al., 2008), insect attack (Kempema et al., 2007; Ehlting et al., 2008), metal toxicity (Weber et al., 2006), and fumigation with sulfur (Zhao and Yi, 2014). Similar transcriptome analyses in Arabidopsis have also been performed at a more basal level to understand physiological functions such as plant defense (Eulgem, 2005; Ditt et al., 2006; Postnikova and Nemchinov, 2012; Weeda et al., 2014), development (Gandotra et al., 2013), and embryogenesis (Li et al., 2007), whereas other studies have investigated transcriptomics in relation to various specific tissues or organs, including pollen (Grennan, 2007; Zou and Yu, 2010), anthers (Feng et al., 2012), floral parts (Zhang et al., 2015), root hairs (Jones et al., 2006), guard cells (Wang et al., 2011), and leaves (Gandotra et al., 2013).

Increasing or decreasing cellular NO levels can affect many important groups of genes including metal-containing enzymes such as peroxidases and catalases (Clarke et al., 2000), various protein kinases (Kumar and Klessig, 2000), receptors (Terrile et al., 2012), and transcription factors such as MYB, HY5, and Trx (Palmieri et al., 2008; Tavares et al., 2014; Zeng et al., 2014). These studies have shown that NO regulates several physiological pathways through intricate translational and transcriptional controls. Therefore, the identification of genes regulated by NO may prove beneficial in incorporating tolerance to multiple stresses in crop plants. In this study, we identified genes that were transcriptionally regulated by NO using a high-throughout RNA-Seq-mediated transcriptomic approach after infiltration of 1 mM S-nitrosocysteine (CysNO) directly into the leaves.

## Materials and methods

### Plant material and CysNO treatment

*Arabidopsis thaliana* accession Col-0 seeds were grown under 16 h of light and 8 h of dark at 18°C. Rosette stage plants (about 4 weeks old) were infiltrated with 1 mM CysNO (mixing equimolar HCL-dissolved l-Cysteine and a sodium nitrite solution) at the abaxial side of the leaf and samples were collected 6 h later into liquid nitrogen. Control plants were infiltrated with buffer (1 mM HCl) only.

### RNA extraction and cDNA synthesis

RNA was extracted from leaf samples (in triplicate) from treated and control plants using RNeasy® Plant Mini Kit (Qiagen) according to the manufacturer's standard protocol. RNA quality was analyzed using an Agilent® 2100 Bioanalyzer (Agilent). Samples (2 μg total RNA) with good RNA Integrity and a 28:12S ratio of 2:1 were then treated with DNase 1. For sequencing, mRNA was then synthesized from 2 μg total RNA using oligo-dT primers. RNA libraries were generated using a TruSeq^TM^ RNA library prep kit (Illumina). Single-stranded cDNA was synthesized through fragmentation and hexamer priming of mRNA to generate double-stranded cDNA libraries. cDNA libraries were quantified using the KAPA library quantification kit (Illumina) and sequenced using a HiSeq-2500 sequencer (Illumina).

### Data analysis

Raw sequencing reads included low-quality reads and adaptors, and so were further processed to obtain high-quality reads. Reads with >10% ambiguous bases or with *Q*20 < 40% were removed (Patel and Jain, 2012). Further, quality control processing was done according to analysis program developed by Theragen ETEX (Korea). High-quality reads were compared to the *A. thaliana* genome using Ensembl (Flicek et al., 2014). Alignment was performed using TopHat (Trapnell et al., 2009) with the default values.

### Measurement of expression levels and identification of differentially expressed genes (DEGs)

Expression levels of various genes were calculated by Cufflinks v2.2.1 (Trapnell et al., 2010) and compared to reference data available in Ensembl. Data were subjected to multi-read-correction and frag-bias-corrections to increase accuracy. Differences in gene expression levels were calculated using Cuffdiff v2.2.1 (Trapnell et al., 2010) to identify DEGs. Genes with value *Q* < 0.05 were selected.

### Annotation and gene ontology analysis

Identified DEGs were further analyzed for annotation and GO terms. For this purpose, annotation and GO search was conducted using NCBI (www.ncbi.nlm.nih.gov) and the Gene Ontology Consortium database (http://www.geneontology.org/). GO enrichment analysis was performed using GO-slim molecular function, biological process, and cellular location. PANTHER overrepresentation test was performed at *P* < 0.05 with *p*-values determined by binomial statistics (Cho and Campbell, 2000).

### MapMan analysis

Substantial data comprising thousands of genes and transcripts often challenges efficient data analysis. Classical GO enrichment analyses based on a database search, though helpful for functional categorization of given gene sets, is less useful if detailed analysis is required at the level of studying genes involved in specific pathways and/or physiological functions. For this purpose, different tools have been developed and used with variable success, e.g., the TM4 suit by Saeed et al. (2003), and GoMiner^TM^ by Zeeberg et al. (2003).

The MapMan-omics data analyses software (Thimm et al., 2004; Usadel et al., 2005; Urbanczyk-Wochniak et al., 2006; http://mapman.gabipd.org) allows visualization of -omics data at the process or pathway level. The software is designed and optimized to map transcriptomic data on currently available databases for many plant species, including *A. lyrata, A. thaliana* (Affymetrix, Agilent, TAIR 6, TAIR 7, TAIR 8, TAIR 9, and TAIR 10)*, Brassica napus, B. rapa, Carica papaya, Citrus, Eucalyptus grandis, Glycine max, Gossypium raimondii, Oryza sativa, Populus trichocarpa, Zea mays*, and many other plant species. MapMan utilizes a hierarchical “BIN”-based ontology system. Specific bins are allocated to biological functions and sub-bins are allocated to individual steps or nodes in that particular biological function in a hierarchical order. For example, BIN number 20 is for stress, BIN number 20.1 is for biotic stress, and 20.2 is for abiotic stress. Similarly, sub-bins for abiotic stress include 20.2.1 (heat stress), 20.2.2 (cold stress), 20.2.3 (drought stress), 20.2.4 (wounding), and 20.2.5 (light). The bin and sub-bin approach minimizes the redundancy usually found in GO enrichment analyses. In addition, the software also utilizes gene expression values and displays the analyzed data as a diagram which enhances comprehension and is of a quality appropriate for presentation. Genes with increased or decreased expression levels are shown as color-coded squares in blocks. This tool has been widely used and constantly evolves to accommodate more plant species and data sets.

In order to get more meaningful information, we analyzed 6436 DEGs through MapMan version 3.6.0RC1 to visualize various genes involved in various pathways and biological functions and their expression patterns. For this purpose, all the data for the genes with significantly differential expression (*P* ≤ 0.05) were arranged in Microsoft Excel with their standard unique locus identifiers and their final expression value [Log2 (FPKM treated/FPKM untreated)] and saved in a tab-delimited format. These files were then mapped against the Arabidopsis “Ath_AGI_LOCUS_TAIR10_Aug2012.m02” database in MapMan. After analyzing the data, selected pathways were combined by adding their respective bins and sub-bins to custom-made images uploaded to MapMan.

### Real-time PCR analysis

In order to validate transcriptomic results and gene transcript levels, real-time PCR was performed for 10 randomly selected genes. For this purpose, leaf samples were collected from control and 1 mM CysNO-infiltrated plants as previously mentioned. Total RNA was extracted using Trizol reagent (Ambion, Life Technologies). cDNA was synthesized from 2 μg total RNA using a DiaStar^TM^ RT Kit (SolGent, Korea). A two-step real-time PCR reaction was performed using an Eco^TM^ real-time PCR system (Illumina) using 2x Quantispeed SYBR Kit (PhileKorea) with 100 ng template DNA and 10 nM each primer in a final volume of 20 μl according to the following protocol: polymerase activation at 95°C for 2 min followed by denaturation at 95°C for 5 s and concurrent annealing and extension at 65°C for 30 s. The Arabidopsis actin (*ACT 2*) gene and a no template reaction were used as controls. Primer sequences are listed in Supplementary Table S8.

## Results

In order to analyze global variations in gene expression during nitrosative stress, leaves of soil-grown Arabidopsis plants were infiltrated with 1 mM CysNO. Similar preliminary experiments were conducted using various concentrations of CysNO and plants grown on both solid Murashige and Skoog (MS) medium and hydroponically. However, we noticed that the artificial growth medium had slight effects on gene expression patterns that were distinct from the effects of CysNO treatment. We also used CysNO concentrations ranging from 0.5 to 2 mM that resulted in mild to extreme effects on plant physiology and gene expression. The 1 mM dose has been used for various NO donors in other experiments, for example, SNP by Polverari et al. (2003) and GSNO by Begara-Morales et al. (2014) and Kneeshaw et al. (2014). Similarly, treatment with 1 mM CysNO has been used in various experiments (Martínez-Ruiz and Lamas, 2004; Lam et al., 2010). Therefore, we decided to use 1 mM CysNO for our experiments and directly infiltrate the abaxial side of leaves for a robust and strong response, and to collect leaf samples 6 h after infiltration to capture rapid changes in the global gene expression profile (The list of 10 most up-regulated and most down-regulated genes has been provided in Table 1 and Table 2, respectively and discussed below in detail).

**Table 1:** **List of top 10 up-regulated genes in the RNA-Seq mediated leaf transcriptome of Arabidopsis inflitrated with 1 mM S-nitrocysteine (CysNO)**.

**Serial no**.	**Accession number**	**FPKM (Val_1) control**	**FPKM (Val_2) 1 mM CysNO**	**Log2 (Val_2/Val_1)**	**Annotation**
1	AT5G05220	0.02000	29.19000	10.86180	Encodes an unknown protein
2	AT1G71520	0.02000	38.18000	10.83590	Encodes a member of the DREB subfamily A-5 of ERF/AP2 transcription factor family. The protein contains one AP2 domain
3	AT4G10290	0.02000	27.65000	10.70240	RmlC-like cupins superfamily protein
4	AT1G51780	0.03000	43.94000	10.41580	Encodes a member of the six Arabidopsis IAA-amino acid conjugate hydrolase subfamily
5	AT2G44810	0.04000	45.12000	10.24670	The DAD1 protein is a chloroplast targeted phospholipase A1 that catalyzes the initial step of jasmonic acid biosynthesis. Mutant has defects in anther dehiscence, pollen maturation, and flower opening
6	AT2G22760	0.04000	44.05000	10.18870	Basic helix-loop-helix (bHLH) DNA-binding superfamily protein
7	AT2G39518	0.06000	61.96000	10.09650	Encodes an unknown protein
8	AT1G22810	0.15000	153.56000	9.96553	Encodes a member of the DREB subfamily A-5 of ERF/AP2 transcription factor family. The protein contains one AP2 domain
9	AT3G11480	0.27000	232.47000	9.76860	Encodes SABATH methyltransferase. It methylates both salicylic acid and benzoic acid. It is highly expressed in flowers, induced by biotic and abiotic stress and thought to be involved in direct defense mechanism
10	AT5G12340	0.10000	90.08000	9.76308	Encodes an unknown protein

**Table 2:** **List of 10 most down-regulated genes in the RNA-Seq mediated leaf transcriptome of Arabidopsis infiltrated with 1 mM S-nitrocysteine (CysNO)**.

**Serial no**.	**Accession number**	**FPKM (Val_1) Control**	**FPKM (Val_2) 1 mM CysNO**	**Log2 (Val_2/Val_1)**	**Annotation**
1	AT4G14819	2.77000	0.03000	−6.71390	Encodes an unknown protein
2	AT2G40610	99.40000	1.58000	−5.97661	Member of Alpha-Expansin Gene Family, involved in the formation of nematode-induced syncytia in roots of *Arabidopsis thaliana*
3	AT2G04460	1.77000	0.03000	−5.94716	Transposable element gene
4	AT1G49200	9.57000	0.17000	−5.84095	Encodes a RING/U-box superfamily protein
5	AT1G23965	1.06000	0.02000	−5.63821	Encodes an unknown protein
6	AT1G77870	5.19000	0.10000	−5.63312	Encodes a membrane-anchored ubiquitin-fold protein 5 precursor (MUB5)
7	AT5G05840	0.48000	0.01000	−5.43983	Encodes an unknown protein
8	AT4G38860	129.68	3.01000	−5.43081	Encodes SAUR-like auxin-responsive family protein
9	AT1G60590	9.60000	0.24000	−5.34142	Encodes an enzyme of the pectin lyase-like superfamily protein
10	AT5G20630	2.94000	0.08000	−5.22176	Encodes a germin-like protein

### Transcriptome analysis

**Figure 1:**
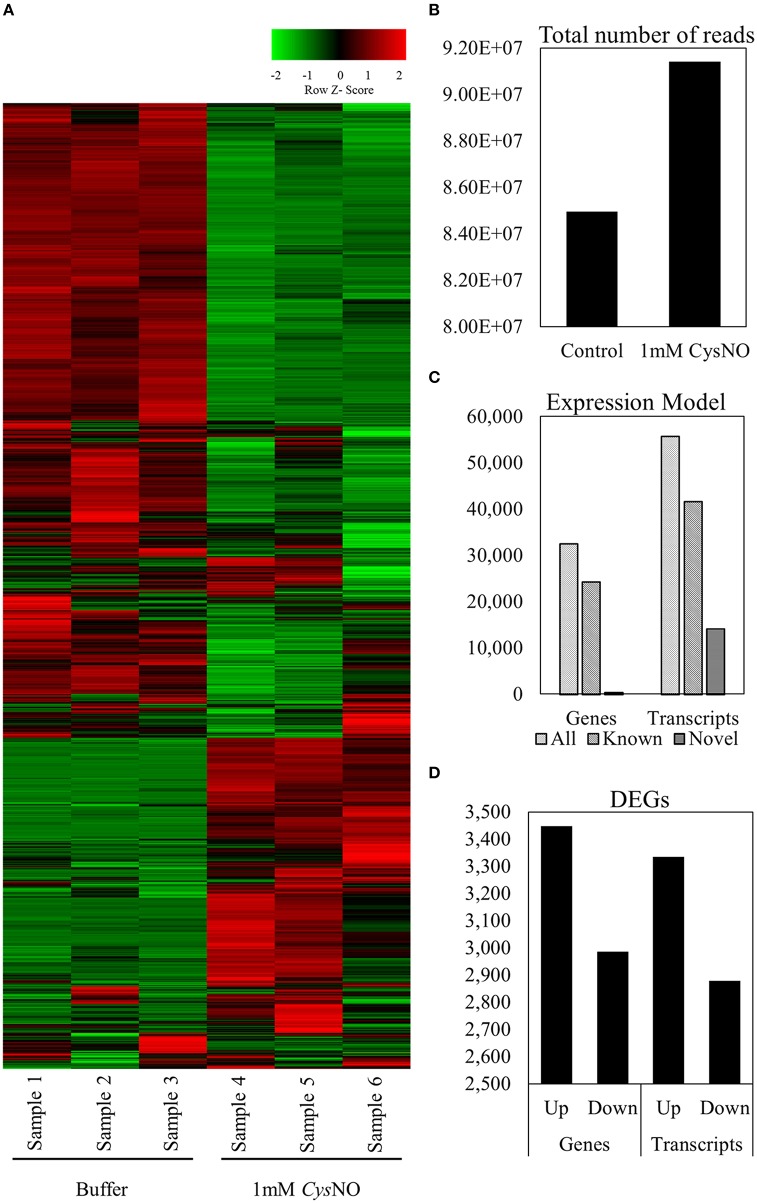
**Arabidopsis leaf transcriptome in response to 1 mM CysNO treatment. (A)** Heat map showing signal intensities of differentially expressed genes in three replicates each of control and CysNO-treated Arabidopsis samples. **(B)** An average of 91,419,167 reads were obtained from samples treated with 1 mM CysNO compared to 84,964,537 reads from control samples. **(C)** More than 95% of all reads were successfully mapped to the Arabidopsis genome, obtaining 32,535 genes (including 24,284 known and 447 novel genes) and 55,753 transcripts (including 41,621 known and 14,132 novel transcripts). **(D)** At a *Q*-value of 0.05, 3444 up-regulated and 2987 down-regulated genes were identified.

An overall representation of the gene expression profile (Figure 1) indicated highly significant changes in expression at a cutoff *Q*-value of 0.05. An average of 91.4 million reads were generated from three biological replicates treated with 1 mM CysNO compared to 84.9 million reads from the control samples (Figure 1). Up to 95% of these reads successfully mapped to the Arabidopsis reference genome (Supplementary Table S1). A total of 32,535 genes (including 24,284 known and 447 novel genes) and 55,753 transcripts (including 41,621 known and 14,132 novel transcripts) were identified (Figure 1). Among these, 3448 genes were up-regulated whereas 2987 were down-regulated by CysNO treatment. Similarly, 3335 and 2879 transcripts were up- and down-regulated, respectively, as a result of CysNO treatment (Figure 1; Supplementary Table S2). After quantile normalization of the FPKM values followed by student's *T*-test at *P* = 0.05 and selection of DEGs with at least 2-folds change in their expression in response to CysNO treatment, we identified 1165 DEGs (463 up-regulated and 702 down-regulated) with highly significant expression patterns before and after the treatment (Supplementary Figure S1). Gene expression and raw sequence data have been submitted to the Gene Expression Omnibus (GEO) and Short Read Archive (SRA) at NCBI (http://www.ncbi.nlm.nih.gov/) with accession numbers GSE81361 and SRP074890, respectively.

### Functional classification of genes: GO-enrichment analysis

**Figure 2:**
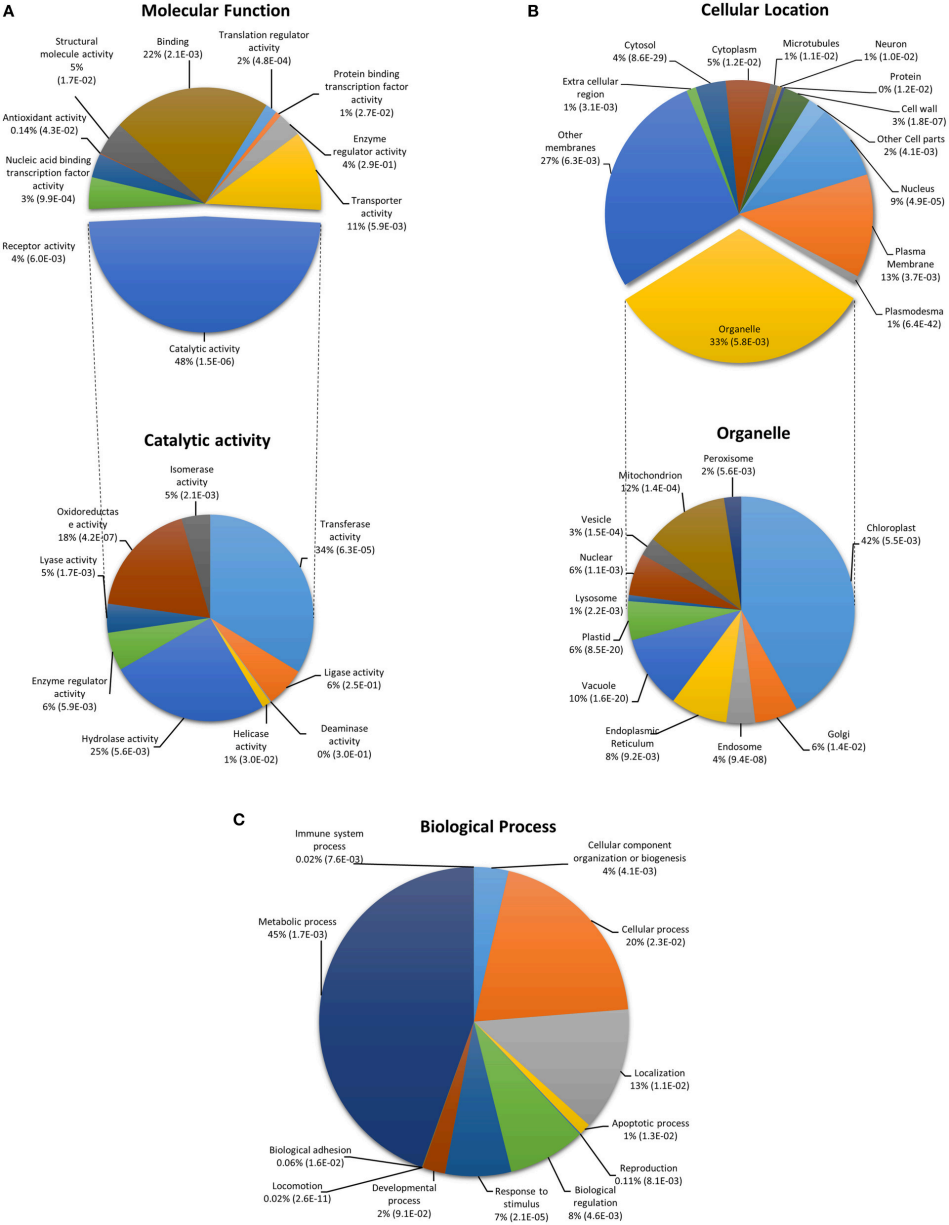
**Functional classification of CysNO-responsive genes in Arabidopsis leaf**. Genes were classified into categories for **(A)** molecular function, **(B)** cellular location, and **(C)** biological process. GO enrichment analysis was performed according to the Gene Ontology Consortium (www.geneontology.org). PANTHER over-representation test was performed through PANTHER (version 10.0) at *P* < 0.05 for PANTHER GO-slim molecular function, cellular location and biological process. The *p*-values shown in brackets are determined by binomial statistics (Cho and Campbell, 2000).

In order to determine whether CysNO-induced genes belong to particular gene classes, gene ontology (GO) classification according to molecular function, biological process, and cellular response was performed. GO enrichment included a broad range of molecular functions, among which catalytic activity (48%), binding activity (22%), and transporter activity (11%) were the dominant categories. Further, detailed analysis within the catalytic activity GO indicated transferase activity (34%), hydrolase activity (25%), and oxidoreductase activity (18%; Figure 2). This finding is consistent with previously published transcriptomic studies demonstrating that binding and catalytic activities operate predominantly at transcriptome level (Lu et al., 2010; Tang et al., 2013). GO terms involving translation regulator and nucleic acid binding activity were also identified in our data set, highlighting the importance of translational activity in transcriptomic responses to nitrosative stress. GO terms for cellular location included membrane-localized genes (40%, including 13% in the plasma membrane and 27% in other membranes) followed by genes related to various cellular organelles (33%), the most abundant of which were related to chloroplast (42%), mitochondrial (12%), and vacuolar proteins (10%; Figure 2). These findings are similar to those of other transcriptomic studies that reported induction of membrane-localized, chloroplast, and vacuole-related genes after stress treatment (Desikan et al., 2001; Begara-Morales et al., 2014). GO terms related to biological processes predominantly included metabolic processes (45%) followed by cellular processes (20%). GO terms involved in biological regulation (8%), stimulus response (7%), and developmental processes (2%) were also abundant (Figure 2).

### DEGs associated with biotic and abiotic stress

All differentially expressed genes (DEGs) were further subjected to functional analysis using MapMan 3.6.0. A total of 787 DEGs were found to be involved in biotic and abiotic stress responses. Of these, 319 were down-regulated while 468 were up-regulated in response to CysNO treatment. Further, functional categorization revealed the presence of membrane-bound receptors [such as AT1G72930, a toll-interleukin receptor-like gene, and AT3G50470, a homolog of *RPW8*, which is involved in the plant basal defense system (Xiao et al., 2003)], pathogenesis-related proteins (including toll-interleukin receptor-like, coiled-coil-nucleotide-binding site-leucine-rich repeat, and protease inhibitor classes), and members of secondary metabolite production pathways (including those responsible for the production of carotenoids, terpenoids, alkaloids, waxes, and phenylpropanoids). Biotic and abiotic stresses are often accompanied by a concurrent change in the cellular redox tone (Foyer and Noctor, 2005). We found several defense-related redox-sensitive DEGs in our transcriptomic data. For example, expression of ascorbate peroxidase 1 (*APX1*-AT1G07890)—which has been shown to be involved in responses to drought (Bhatt et al., 2013), metal toxicity (Fourcroy et al., 2004), and heat (Storozhenko et al., 1998)—doubled, whereas that of thioredoxin 5 (*TRX5*-AT1G45145) increased by more than 30-times. Among other DEGs from the thioredoxin family, glutathione synthetase 2 (*GSH2*-AT5G27380) was up-regulated 4-times whereas the expression of catalase 2 (*CAT2*-AT4G35090) and catalase 3 (*CAT3*-AT1G20620) was reduced by more than 2-times. Differential expression patterns of several other members of the oxidoreductase and glutaredoxin families of proteins were also recorded (Supplementary Figure S2; Supplementary Table S3). Published research on Arabidopsis and other plants shows the involvement of catalase genes in biotic and abiotic stresses (Du et al., 2008; Mhamdi et al., 2010; Li et al., 2015). Similarly, NO-responsive genes involved in other abiotic stress responses were also found in our transcriptomic data, e.g., heat shock proteins, low temperature-responsive proteins, and drought-responsive proteins, especially members of the Early Response to Dehydration (ERD) class of proteins.

### DEGs involved in hormone metabolism

The role of NO in hormone signaling is well-known, including in relation to post-translational modifications of hormone-responsive transcription factors (Hill, 2015) and auxin receptor TIR1 (Terrile et al., 2012). Indeed, substantial cross-talk exists between NO and phytohormone signaling (Sanz et al., 2015). Functional annotation of our transcriptomic data identified GO terms involved in hormonal activity. A total of 199 DEGs involved in hormone metabolism were either up- or down-regulated as a result of CysNO treatment. These DEGs were involved in biosynthesis, degradation, and signaling of phytohormones, such as abscisic acid (ABA), auxin (IAA), cytokinin, gibberellic acid (GA), and the defense hormones jasmonic acid (JA) and salicylic acid (SA). Detailed analysis indicated that critical enzymes in the ABA pathway, such as *KAO1* (AT1G05160; a member of the cytochrome P450-88A3 family that encodes an ent-kaurenoic acid hydroxylase), *NCED4* (AT4G19170; encoding epoxycarotenoid dioxygenase 4), and ABA-Deficient 2 (*ABA2*-AT1G52340; encoding xanthoxin dehydrogenase) are down-regulated. Conversely, *NCED3* (AT3G14440) and *AAO3* (AT2G27150; encoding abscisic acid aldehyde oxidase 3) were up-regulated by 13 and 3-folds, respectively.

*KAO1* is also an important component of the GA metabolic pathway, in which it converts ent-kaurenoic acid to GA12 aldehyde. Expression of two homologs (AT1G02400 and AT1G30040) that encode GA2 oxidases increased by more than 300 and 10-folds, respectively. The well-known SA pathway-regulating enzymes isochorismate synthase (*ICS1*-AT1G74710 and *ICS2*-AT1G18870) and SA-glucosyl transferase (*SAGT*-AT2G43820) were up-regulated by at least 2-times, whereas the expression of *BSMT1* (AT3G11480) increased by more than 800-folds. ICS1 is required for SA synthesis and plant defense against bacterial pathogens (Wildermuth et al., 2001). Similarly, a significant increase in the expression of key jasmonate signaling pathway regulatory enzymes was observed. For example, transcript accumulation of defective anther dehiscence 1 (*DAD1*-AT2G44810) increased by more than 1000x; accumulation of lipoxygenase 3 (*LOX3*-AT1G17420), allene oxide cyclase 3 (*AOC3*-AT3G25780), and oxophytodienoate reductase 3 (*OPR3*-AT2G06050) transcripts increased by 100x, accumulation of *AOC1* (AT3G25760), *AOC2* (AT3G25770), and *OPR1* (AT1G76680) transcripts increased by 50x; and accumulation of allene oxide synthase (*AOS*-AT5G42650) and *OPR2* (AT1G76690) transcripts increased 10-times (Supplementary Figure S3; Supplementary Table S4).

NO plays an important role in drought tolerance by regulating ABA-mediated stomatal movement (Shi et al., 2015; Xu et al., 2015) and also interacts with GA to regulate root growth (Wu et al., 2014), chilling tolerance (Li et al., 2013), and response to metal toxicity during seed germination (Zhu et al., 2012). Expression of ICS1 in response to bacterial pathogens has been shown to increase with a concomitant rise in SA levels especially levels of SA-glucoside potentiated by *SAGT* (Ding et al., 2015). Previous reports also suggest that treatment of Arabidopsis plants with SNP strongly induces the expression of AOS and LOX2 (Huang et al., 2004). Studies concerning the interaction of NO with phytohormones relating to defense (SA and JA) was reviewed by Mur et al. (2013), and the interaction of NO with other phytohormones was reviewed by Freschi (2013) and París et al. (2013).

### DEGs associated with secondary metabolites

Plant secondary metabolites are not required for plants to survive but aid in growth, development, and primary metabolism. Accepted roles for various plant secondary metabolites include plant defense against pathogens and herbivores (Maag et al., 2015) and their use as pesticides (Cespedes et al., 2015). However, the molecular mechanisms underlying their roles in plant biology are largely unknown, and the concept that secondary metabolites are not required for plant survival somewhat undermines the perception of their importance. NO is known to regulate elicitor-dependent and elicitor-independent production of pharmaceutically important secondary metabolites [reviewed by Zhang et al. (2012)].

We intended to elucidate the effects of CysNO treatment on Arabidopsis secondary metabolite fluxes through our transcriptomic analysis. Analysis of GO terms relating to secondary metabolism were identified 298 DEGs in response to CysNO treatment. These DEGs were involved in metabolism of a variety of secondary metabolites, including flavonoids (such as chalcones, isoflavonoids, flavonols, dihydroflavonols, and anthocyanins), glucosinolates, phenylpropanoids, various lignins, tocopherol, carotenoids, terpenoids, and various steps of the shikimate pathway. A detailed list of these genes along with their description and expression values is given in Supplementary Table S5. Briefly, detailed investigation of DEG involvement in various steps of secondary metabolite homeostasis revealed CysNO-induced up-regulation of key enzymes by at least 10-folds e.g., 3-deoxy-d-arabino-heptulosonate 7-phosphate synthase 1 (*DHS1*-AT4G39980) and 5-enolpyruvylshikimate 3-phosphate synthase (*EPSP* synthsae-AT2G45300), which is involved in the shikimate pathway (Supplementary Figure S4).

Genes involved in the tocopherol biosynthesis pathway, the products of which often exhibit vitamin E activity, were mostly down-regulated. These included Vitamin-E Deficient 1 (*VTE1*-AT4G32770), which encodes a tocopherol cyclase that is required for α-tocopherol synthesis, and γ-tocopherol methyl transferase (*VTE4*-AT1G64970), which is required for β-tocopherol synthesis.

Similarly, all DEGs in the carotenoid pathway except carotenoid isomerase (*CrtISO*-AT1G06820), which was up-regulated by 30-folds, were down-regulated in response to CysNO treatment. These included lycopene β-cyclase (*LCYb-*AT3G10230), phytoene synthase (*PSY*-AT5G17230), zeta-carotene desaturase (*ZDS-*AT3G04870), and two isoforms of violaxanthin de-epoxidase (*VDE-*AT1G08550 and AT2G21860).

In the phenylpropanoid and flavonoid pathways, expression of two isoforms of the Arabidopsis phenylalanine ammonialyase (*ATPAL1*-AT2G37040 and *ATPAL2*-AT3G53260), which are responsible for the conversion of phenylalanine to cinnamic acid, more than doubled. Relatedly, five isoforms of 4-coumarate-CoA ligase (4CL) that catalyze multiple steps in the synthesis of various phenylpropanoids were differentially expressed in response to CysNO treatment. Among these, *4CL1* (AT1G51680), *4CL2* (AT3G21240), and *4CL5* (AT3G21230) were up-regulated 5, 10, and 80-folds, respectively. Similarly, two isoforms of the multi-step Arabidopsis cinnamyl alcohol dehydrogenase, *CAD1* (AT1G72680) and *CAD5* (AT4G34230), were up-regulated by 5 and 30x, respectively. Finally, ferulate 5-hydroxylase (*5FH*-AT5G04330) and caffeate O-methyl transferase (*COMT*-AT5G54160) were each up-regulated 5-times and expression of dihydroflavonol 4-reductase-*DFR* (AT5G42800) was up-regulated 3-times (Supplementary Figure S4; Supplementary Table S5).

### DEGs with GO terms involved in cellular functions

Experiments utilizing various NO donors show modulation of a plethora of cellular functions. Analysis of our transcriptomic data for GO terms related to cellular function revealed differential expression of several genes that play essential roles in cell division and cell cycle, DNA repair, vesicle transport, and protein targeting, RNA processing, cellular redox tone, metal handling, and protein modifications. Transport is perhaps one of the oldest research topics in plant physiology and has been studied in detail at the cellular, organismal, and whole-plant level. A key component of cellular transport is the movement of molecules in and out of the cell across membranes, a process that is mediated by both active and passive transport systems. Changes in transportation schedules is one of the important events following stress induction. Many DEGs that encode cellular transporters were detected (Supplementary Figure S5; Supplementary Table S6).

DEGs responsible for the transport of nutrients such as nitrate and ammonium included three members of the well-known high-affinity nitrate transporter (NRT2) family (Glass et al., 2002), specifically *NRT2.6* (AT3G45060), *NRT2.7* (AT5G14570), and *NRT1.*1 (AT1G12110). A significant increase in their expression was observed, especially that of *NRT2.6*, which increased 70-folds. In addition, expression of the wound-responsive transmembrane transporter *WR3* (AT5G50200) was up-regulated by 10x. DEGs responsible for transport included seven calcium cation exchangers (CAX), four of which (*CAX1-*AT2G38170*, CAX2-*AT3G13320*, CAX3*-AT3G51860, and putative *CAX10-*AT1G54110) were slightly down-regulated, whereas the remaining three (*CAX7*-AT5G17860, *CAX8*-AT5G17850, and *CAX9*-AT3G14070) were up-regulated 8, 30, and 6-folds, respectively. These cation exchanger antiporters comprise a group of proteins responsible for shuttling various ions across the cell membrane in order to maintain biological equilibrium (Hirschi, 2004).

Plant potassium transporters can be broadly classified into three categories: potassium ion permeases, potassium ion transporters, and cation proton antiporters (Gierth and Mäser, 2007). Potassium transport-related DEGs included hyperpolarization-activated inward-rectifier K^+^ channels comprising Arabidopsis *AKT1* (AT2G26650; up-regulated 7) and *AKT2* (AT4G22200; down-regulated 2-folds), which are responsible for controlling potassium influx (Rodríguez-Navarro and Rubio, 2006), and the depolarization-activated outward-rectifier K^+^ channels comprising Arabidopsis *KCO5* (AT4G01840; up-regulated 2-folds) and *KCO6* (AT4G18160; up-regulated by 2x), which control potassium efflux (Pei et al., 1998).

Similarly, other active-transport systems remove unwanted or toxic molecules from the cell. Examples of such an efflux system include drug-specific or multidrug and toxin extrusion (MATE) systems and ATP binding cassette (ABC) transporters. Hundreds of these transporters have been characterized in prokaryotes (Choi, 2005). Analysis of our transcriptomic data revealed the presence of 42 genes encoding ABC transporter and multidrug resistance proteins. For example, activated disease susceptibility 1 (*ADS1*-AT4G29140), which encodes a MATE protein, was up-regulated. This gene has been shown to negatively regulate plant disease resistance and the *ads1*-dominant knockout mutant has been shown to accumulate higher levels of reactive oxygen intermediates, such as H_2_O_2_, in an RBOH-dependent manner (Sun et al., 2011). Similarly, the Arabidopsis detoxification efflux carrier-MATE transporter (*DTX50*-AT5G52050), which was up-regulated by more than 20x in response to 1 mM CysNO treatment, is a membrane-localized ABA efflux transporter (Zhang et al., 2014).

Natural compounds secreted by plant roots influence the surrounding soil microbial diversity. One of these, the Arabidopsis ABC transporter pleotropic drug resistance 2 (*PDR2-*AT4G15230), was up-regulated by at least 30x. An Arabidopsis *pdr2* loss-of-function mutant exhibited alterations in root exudates and as a result significantly altered fungal and bacterial diversity in the rhizosphere (Badri et al., 2009). Similarly, other DEGs encoding various transporters were abundant in our dataset. A detailed list of all these genes and their expression values is presented in Supplementary Table S6.

### DEGs associated with transcriptional regulation

We found 604 DEGs (274 down-regulated and 330 up-regulated) encoding known and putative transcription factors (TFs) of various classes. We found 44 AP2/AREBP TFs (7.28%), 8 ARF TFs (1.3%), 14 Aux/IAA TFs (2.317%), 55 BHLH TFs (9.1%), 70 ZNF TFs (11.7%), 19 HB TFs (3.14%), 53 MYB TFs (8.7%), 34 WRKY TFs (5.6%), 25 bZIP TFs (4.31%), 27 putative TFs (4.47%), and 78 (12.91%) unclassified TFs. A list of these transcription factors, their descriptions, and expression values can be found in Supplementary Figure S6; Supplementary Table S7. Here, we describe selected differentially expressed TFs per available information in the literature. Expression of the AP2/EREBP TFs *RAP2.4* (AT1G78080) and *RAP2.4B* (AT1G22190), both encoding abiotic stress-associated dehydration-responsive element binding factor (DREB) A-6 clade proteins, increased by more than 3x. Both of these proteins localize exclusively in the nucleus and have similar binding characteristics within their dehydration-responsive elements (DREs). Microarray analysis of Arabidopsis double knockout and overexpression plants for these two genes indicated variation in the expression profiles of drought-related gene sets, thus indicating the involvement of these two TFs in drought tolerance (Rae et al., 2011). Expression of the *RAP2.6* (AT1G43160), which encodes an ERF/AP2 TF, increased by more than 800x in response to 1 mM CysNO. This gene has been shown to positively regulate the expression of JA-responsive genes in response to infection by *Heterodera schachtii* as Arabidopsis plants over-expressing this gene deposited increased callose in the cynctia (Ali et al., 2013). Expression of the Dwarf and Delayed Flowering 1 (*DDF1*-AT1G12610) AP2/EREBP TF, which encodes a member of the CBF/DREB1 subfamily, increased by more than 70x. Arabidopsis plants overexpressing this TF exhibited enhanced tolerance to freezing temperatures, heat, drought (Kang et al., 2011), salinity, and reduced bioactive GA (Magome et al., 2008). TFs like ABA responsive 1 (*ABR1*-AT5G64750), ABA-inducible BHLH (*AIB*-AT2G46510), and ABA insensitive 5 (*ABI5*-AT2G36270) were up-regulated by 800, 6, and 10-folds, respectively. AB15, a leucine zipper TF, controls ABA-dependent repression of growth after germination (Lopez-Molina et al., 2002). It was recently shown that this ABI5-mediated growth regulation is only possible due to the effects of NO, as S-nitrosylation of ABI5 at Cys-153 enables its degradation through CULLIN4 and KEEP ON GOING (KOG) E3 ligases and thus promotes seed germination and growth (Albertos et al., 2015).

Expression of the transcriptional repressor TGA2 (AT5G06950) was reduced by 2x in response to CysNO treatment. TGA2 and NPR1 are both activators of systemic acquired resistance (SAR). At basal levels, TGA2 represses NPR1 overexpression. After induction of SAR by a pathogen, the BTB/POZ domain of NPR1 interacts with and abolishes the repressive effect of TGA2 by removing TGA2 oligomers from the target DNA (Boyle et al., 2009). Trade-off between the Aux/IAA auxin-responsive transcriptional repressors and ARF transcriptional activators is important for normal auxin-dependent responses in Arabidopsis. The expression of transcriptional repressors Aux/IAA 2-11 (AT5G43700), Aux/IAA 14 (AT4G14550), and Aux/IAA 29 (AT4G32280) was reduced by 9, 11, and 28x, respectively, in response to treatment with 1 mM CysNO. Aux/IAA repressors have been shown to bind ARF transcriptional activators. After ubiquitin-dependent degradation of these repressors, ARF are released to activate auxin responsive genes (Nanao et al., 2014). Interestingly, two ARF-encoding genes, *ARF6* (AT1G30330) and *ARF5* (AT1G19850), were up-regulated by at least 4x.

Many DEGs that regulate the ubiquitin- and autophagy-dependent degradation machinery were also identified. Of these, 73 DEGs were involved in ubiquitin::E3::SCF::FBOX complexes and two were involved in SKP complexes. Similarly, other TFs involved in disease resistance, ABA, GA, auxin, SA, JA, NO, and H_2_O_2_ signaling were abundant in our data set (Supplementary Table S7).

### Confirmation of CysNO-mediated transcriptional changes by quantitative RT-PCR

**Figure 3:**
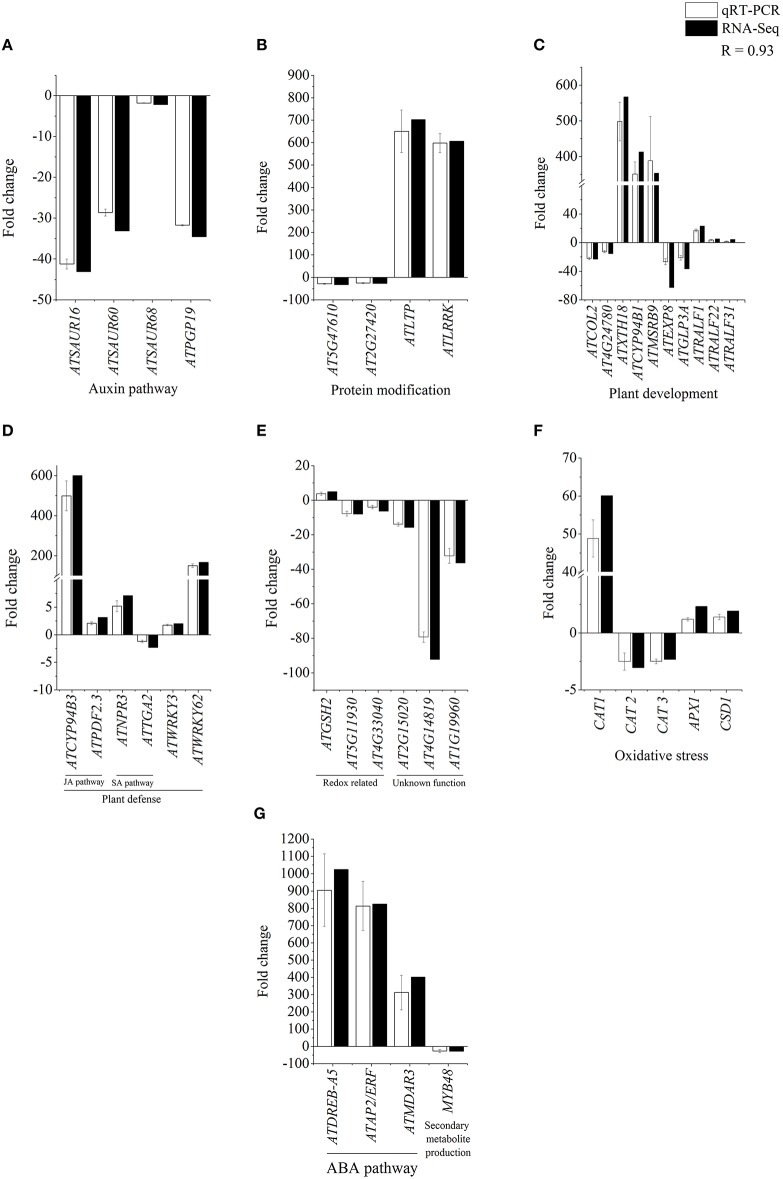
**Confirmation of RNA-Seq results by qRT-PCR analysis**. Total 39 genes identified as CysNO-responsive through RNA-Seq-based transcriptomic analysis (black bars) in Arabidopsis leaves were selected to validate the fold change in their expression values through qRT-PCR analysis (white bars). These genes belonged to different functional groups involved in the regulation of auxin pathway **(A)**, protein modification **(B)**, plant development **(C)**, plant defense **(D)**, redox related processes **(E)**, oxidative stress **(F)**, ABA pathway and secondary metabolite production **(G)**. As indicated by a correlation coefficient (*R*) of 0.93, RNA-Seq and qRT-PCR results are significantly comparable to each other, and RNA-Seq results are reliable. Error bars indicate the standard deviation and each point represents the mean of three replicates.

**Figure 4:**
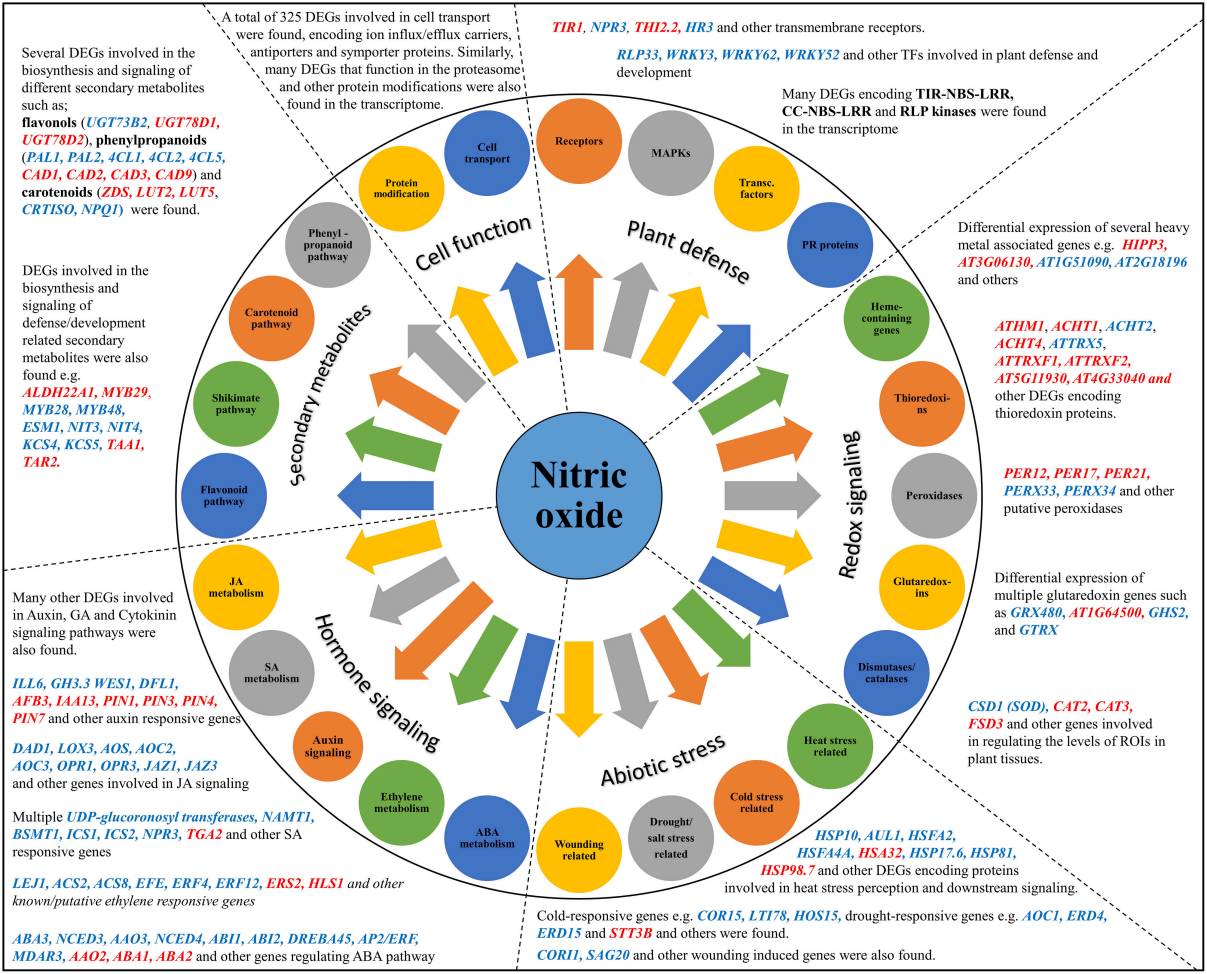
**Model summarizing the different regulatory pathways and their respective DEGs in the Cys-NO responsive transcriptome of Arabidopsis**. GO enrichment analysis and functional classification identified DEGs (up-regulated blue font, down-regulated red font) involved in important physiological activities such as; plant defense and development, redox stress signaling, abiotic stress regulation, hormone signaling, secondary metabolite production, protein modification, cell transport, and many others. Representative genes from each pathway were chosen for validation of their expression pattern through quantitative real time PCR analysis.

In order to further validate CysNO-induced transcriptional changes, 39 different genes were chosen from different functional categories/pathways (**Figure 4**) to determine changes in their transcript accumulation 6 h after infiltration with 1 mM CysNO infiltration (Figure 3). These genes were involved in functional pathways such as IAA, ABA, and JA signaling, protein modification, redox homeostasis, oxidative stress perception, plant defense, and development. Relatively higher variation in expression patterns between qRT-PCR and transcriptomic data was found only for those genes that exhibited very low basal level expression, e.g., *ATEXP8* and *AT4G14819* (Figure 3). However, a correlation coefficient of 0.93 shows that the RNA-Seq results were statistically reliable and comparable to transcriptomic data.

## Discussion

The results of the transcriptome analysis are summarized in the form of a model showing the major functional pathways and DEGs activated after CysNO treatment (Figure 4). NO is now recognized as a major signaling molecule in eukaryotes, including plants. NO is generated in plants under non-stressful conditions as well as conditions induced by a variety of factors. CysNO has been widely used as a NO donor in various studies. Its low molecular weight enables easy and rapid diffusion into the plant systems when compared to other NO donors. Intricate cross-talk and tradeoffs of NO have been found with other types of stress signals, especially H_2_O_2_ (Dubovskaya et al., 2007; Sang et al., 2008a; Tanou et al., 2009, 2010), as mediated through strict transcriptional and translational control. This makes it clear that the identification of such transcriptional changes would aid elaboration of the mechanistic control of various functions. However, a global-scale analysis of NO-regulated transcriptional changes has not yet been described.

We therefore performed a large-scale sequence analysis spanning more than 176 million reads to characterize nitrosative stress responses at the transcriptome level, and identified 3448 up-regulated and 2987 down-regulated genes using a *Q*-value of 0.05. The MDS and PCA plots showing these DEGs are presented in Supplementary Figure S7. Among these, more than 94% of genes showed higher than 2-fold change in expression following CysNO treatment. We performed prior experiments with different doses of CysNO using various application methods and identified and optimized a strategy of direct infiltration with 1 mM CysNO for a robust and rapid transcriptional response, as indicated by the high number of DEGs and the resulting high variation in gene expression profiles when compared to other stress-induced transcriptomic analyses in Arabidopsis. Consequently, smaller transcriptional variations can lead to larger translational consequences. We successfully mapped more than 95% of all the reads to the Arabidopsis genome. The remaining 5% of mismatched data may represent a promising source for discovery of novel genes.

Changes in cellular concentrations of NO and its derivatives affect the redox state of the cell. We found a high number of DEGs responsible for mediating counter-responsive measures in response to increasing nitrosative stresses. We found multiple members of the thioredoxin, heme-binding protein families, disulfide isomerases, mono- and di-hydro ascorbate reductases, and glutathione peroxidases. Such a counter-response was also evident from the very high catalytic activity observed (Figure 2), highlighting the post-nitrosative-stress cellular environment with hyperactive physiological processes, especially total oxidoreductase activity. A global equilibrium between RNS and ROS is required for normal physiological functions at the cellular level. Following infiltration of 1 mM CysNO into the Arabidopsis leaf apoplast, expression of the two predominant catalase enzymes, *CAT2* and *CAT3*, decreased after CysNO treatment and which would ultimately lead to increased cellular H_2_O_2_ levels in order to achieve a balance between cellular nitrogen and oxygen species. We also found substantial induction of genes involved in oxidative pathways, such as *APX1, RBOH-D*, and *RBOH-F* (Supplementary Figure S2), which helped counter the increasing nitrosative stress.

Certain cellular compartments are required in these counter-responsive measures. Out of the 33% DEGs with GO terms related to different organelles, 42% were found to be related to the chloroplast followed by 12% to the mitochondria and 10% to the vacuole (Figure 2). DEGs related to these organelles have also been identified found by other stress-induced transcriptomic analyses (Desikan et al., 2001; Begara-Morales et al., 2014). Endogenous NO synthesis involves multiple pathways arising in different organelles such as chloroplasts, which are a key proposed site for NO production in plants (Jasid et al., 2006; Galatro et al., 2013; Tewari et al., 2013), mitochondria (Gupta et al., 2009), and peroxisomes (Corpas et al., 2009). Other studies have also established a major role for chloroplasts in endogenous NO production under basal and stressed conditions (Foissner et al., 2000; Gould et al., 2003; Arnaud et al., 2006), as several components of the electron transport chain in the photosystem-II have been identified as potential targets of NO in the chloroplast (Petrouleas and Diner, 1990). From experiments using different NO donors we know that the effect of NO on the chloroplast is mostly dose-dependent. The targets and actions of NO in the chloroplast are reviewed by Misra et al. (2014). This, along with the high number of DEGs expressed in the chloroplast, indicates the importance of this organelle in NO biology, and the chloroplastic DEGs in our dataset may serve as a good source for identification of NOS-like enzyme(s) directly or indirectly involved in NO biosynthesis.

The highly important plant defense-related enzyme benzoic acid/SA carboxyl methyltransferase (BSMT1-AT3G11480) was up-regulated by more than 800x, although basal expression levels in the control samples were very low. BSMT1 is responsible for the production of methyl salicylate (MeSA) from SA, as Arabidopsis *bsmt1* knockout plants cannot accumulate MeSA and therefore cannot exhibit SAR (Liu et al., 2010). This means that after exogenous application of NO, a robust increase in expression of *BSMT1* would result in increased MeSA levels, thus establishing strong SAR responses; as has been shown that exogenous MeSA application results in increased NO content in plants (Zhang et al., 2011). DEGs involved in biotic and abiotic stresses included genes responsible for the production of secondary metabolites, including various lignans that are required for membrane reinforcement as a protective strategy against stress signals.

We also found 60 DEGs (43 up-regulated and 17 down-regulated) encoding known and putative calcium-binding calmodulin proteins, indicating the involvement of calmodulin proteins in stress tolerance (Supplementary Table S2). Significant crosstalk exists between the signaling pathways of H_2_O_2_, calcium-calmodulin, and NO. NO Accumulation decreases in the presence of calmodulin antagonists, whereas SNP application increases Ca^2+^ concentration and calmodulin 1 (*CAM1*) expression in maize (Sang et al., 2008b).

Most DEGs that increased in expression by 100–1000x after CysNO treatment were found to have very low expression in control samples, and most do not appear to have a direct link to nitrosative stress. However, the role of these DEGs in other types of stresses, such as oxidative, biotic, and abiotic stress, might explain their sensitivity to CysNO (Supplementary Table S2). These genes had a range of potential functions when analyzed for GO enrichment. Several DEGS encoded heat shock proteins. Out of these, six belonged to the heat shock transcription factor family, of which five were up-regulated whereas one was slightly down-regulated. NO has been shown to play important role in heat stress physiology in plants (Lee et al., 2008) and its cross-talk with various ROS establishes thermo-tolerance through the expression of heat shock proteins, as in the case of Arabidopsis seedlings (Wang et al., 2014; Wu et al., 2015).

Mitogen-activated protein kinase (MAPK) signaling regulates the production of NO in *Nicotiana benthamiana* (Asai et al., 2008). We identified 14 DEGs that encode MAPKs. MPK6 interacts physically with and phosphorylates the nitrate reductase enzyme NIA2 at Ser-627, leading to increased nitrate reductase activity as both NO concentration and nitrate reductase activity dramatically decreased in *mpk6* mutants in response to H_2_O_2_ (Wang et al., 2010). We also observed increased expression of several MAPK phosphatases. For example, AT2G30030 (encoding AP2C1 protein phosphatase) and AT2G40180 (encoding PPC2-5 phosphatase) were up-regulated by 2 and 50x, respectively, after CysNO treatment. These two genes have shown to negatively regulate MPKs through dephosphorylation (Umbrasaite et al., 2011). The resulting suppression of MPKs could lead to reduced nitrate reductase activity, and this may also be one of the reasons that we did not detect any of the two nitrate reductase enzymes in our transcriptomic data, as it is likely more important to reduce endogenous NO production following exogenous CysNO application.

We also identified 21 DEGs involved in metal handling, such as the heavy metal-associated isoprenylated plant proteins (HIPP). *HIPP21* (AT5G17450), *HIPP25* (AT4G35060), and *HIPP26* (AT4G38580) were up-regulated by more than 2x following CysNO treatment. Expression of the Arabidopsis *CUTA* gene (AT2G33740), which encodes a chloroplast-related copper binding protein (Burkhead et al., 2003), decreased by more than 2x. The plant-specific HIPP family contains key proteins responsible for cellular metal transport, drought tolerance, and disease resistance (de Abreu-Neto et al., 2013). Arabidopsis phytochelatin synthase (*PCS1-*AT5G44070) was up-regulated by nearly 10x (Supplementary Table S2). This enzyme catalyzes the synthesis of phytochelatin from glutathione at basal levels and in the presence of metal ions like Cd^2+^, Zn^2+^, Cu^2+^, and Fe^3+^. It has been recently used to engineer heavy metal tolerance in plants (Cahoon et al., 2015) and to engineer cadmium biosensers in yeast (Matsuura et al., 2013), and its over-expression in tobacco has been shown to enhance arsenic and cadmium detoxification (Zanella et al., 2015). However, the ubiquitous nature and constitutive expression of phytochelatin synthases suggest a rather more generalized function besides metal detoxification, as *PCS1* was later shown to be involved in non-host resistance (Clay et al., 2009). The nearly 10x increase in its expression after CysNO treatment and its interaction with glutathione for the synthesis of phytochelatin indicates the importance of NO in heavy metal detoxification.

A broad analysis of DEGs in our (and other post-stress) transcriptomic data reveals a counter-responsive model by which plants respond to these stresses. Key components of this model adapted for nitrosative stress includes activation of a broad range of enzymatic activities, involving oxido-reductive reactions, with the aim of returning the cellular redox state back to a physiological normal. This is accompanied by rapid mobilization of the transportation machinery, as increased metabolic activity would require the supply and removal of different components. This in turn requires fine-tuning of the entry and exit checkpoints at cellular membranes, as is evident from the very high number of membrane-localized DEGs that were identified. We found a relatively large number of DEGs expressed in the vacuole along with those involved in the E3 ligase-ubiquitin protein degradation machinery, thus indicating that efficient compartmentalization, packing, and processing are also important in reliable stress-responses. We identified 78 DEGs involved in regulation of photosystem I, photosystem II, electron transport, and the Calvin cycle and, interestingly, all were down-regulated. This shows that stress responses, being exhaustive, and energy-costly, may cause a reduction in growth-related processes. In addition, NO-induced responses include transcriptional changes of a large array of gene sets, many of which are shared by other stress-responses, such as oxidative, drought, salt, heat, cold, and biotic stress, such as pest or pathogen attack. Collectively, the transcriptomic data generated in this study will serve as a good platform for further studies investigating NO biology in plants.

## Author contributions

AH, BY conceived the original screening and research plans, BM, QI, SL, TA, and MS performed the experiments, AH and BM analyzed the data, AH, KK, and BY prepared the manuscript.

## Funding

This work was supported by a grant from the Next-Generation BioGreen 21 Program (SSAC, Grant No. PJ01110201), Rural Development Administration, Republic of Korea.

### Conflict of interest statement

The authors declare that the research was conducted in the absence of any commercial or financial relationships that could be construed as a potential conflict of interest.

## References

[B1] Albertos P. Romero-Puertas M. C. Tatematsu K. Mateos I. Sanchez-Vicente I. Nambara E.. (2015). S-nitrosylation triggers ABI5 degradation to promote seed germination and seedling growth. Nat. Commun. 6:8669. 10.1038/ncomms9669 26493030 PMC4639896

[B2] Ali M. A. Abbas A. Kreil D. P. Bohlmann H. (2013). Overexpression of the transcription factor RAP2.6 leads to enhanced callose deposition in syncytia and enhanced resistance against the beet cyst nematode *Heterodera schachtii* in Arabidopsis roots. BMC Plant Biol. 13:47. 10.1186/1471-2229-13-47 23510309 PMC3623832

[B3] Arnaud N. Murgia I. Boucherez J. Briat J. F. Cellier F. Gaymard F. (2006). An iron-induced nitric oxide burst precedes ubiquitin-dependent protein degradation for Arabidopsis *AtFer1* ferritin gene expression. J. Biol. Chem. 281, 23579–23588. 10.1074/jbc.M602135200 16782706

[B4] Asai S. Ohta K. Yoshioka H. (2008). MAPK signaling regulates nitric oxide and NADPH oxidase-dependent oxidative bursts in *Nicotiana benthamiana*. Plant Cell 20, 1390–1406. 10.1105/tpc.107.055855 18515503 PMC2438462

[B5] Badri D. V. Quintana N. El Kassis E. G. Kim H. K. Choi Y. H. Sugiyama A.. (2009). An ABC transporter mutation alters root exudation of phytochemicals that provoke an overhaul of natural soil microbiota. Plant Physiol. 151, 2006–2017. 10.1104/pp.109.147462 19854857 PMC2785968

[B6] Barouch L. A. Harrison R. W. Skaf M. W. Rosas G. O. Cappola T. P. Kobeissi Z. A.. (2002). Nitric oxide regulates the heart by spatial confinement of nitric oxide synthase isoforms. Nature 416, 337–340. 10.1038/416337a 11907582

[B7] Begara-Morales J. C. Sánchez-Calvo B. Luque F. Leyva-Peréz M. O. Leterrier M. Corpas F. J.. (2014). Differential transcriptomic analysis by RNA-seq of GSNO-responsive genes between Arabidopsis roots and leaves. Plant Cell Physiol. 55, 1080–1095. 10.1093/pcp/pcu044 24599390

[B8] Bhatt D. Saxena S. C. Jain S. Dobriyal A. K. Majee M. Arora S. (2013). Cloning, expression and functional validation of drought inducible ascorbate peroxidase (*Ec-apx1*) from *Eleusine coracana*. Mol. Biol. Rep. 40, 1155–1165. 10.1007/s11033-012-2157-z 23065288

[B9] Bolwell G. P. (1999). Role of active oxygen species and NO in plant defence responses. Curr. Opin. Plant Biol. 2, 287–294. 10.1016/S1369-5266(99)80051-X 10459001

[B10] Boyle P. Le Su E. Rochon A. Shearer H. L. Murmu J. Chu J. Y.. (2009). The BTB/POZ domain of the Arabidopsis disease resistance protein NPR1 interacts with the repression domain of *TGA2* to negate its function. Plant Cell 21, 3700–3713. 10.1105/tpc.109.069971 19915088 PMC2798319

[B11] Burkhead J. L. Abdel-Ghany S. E. Morrill J. M. Pilon-Smits E. A. Pilon M. (2003). The Arabidopsis thaliana *CUTA* gene encodes an evolutionarily conserved copper binding chloroplast protein. Plant J. 34, 856–867. 10.1046/j.1365-313X.2003.01769.x 12795705

[B12] Cahoon R. E. Lutke W. K. Cameron J. C. Chen S. Lee S. G. Rivard R. S.. (2015). Adaptive engineering of phytochelatin-based heavy metal tolerance. J. Biol. Chem. 290, 17321–17330. 10.1074/jbc.M115.652123 26018077 PMC4498070

[B13] Cespedes C. L. Alarcon J. E. Aqueveque P. Seigler D. S. Kubo I. (2015). In the search for new secondary metabolites with biopesticidal properties. Isr. J. Plant Sci. 62, 216–228. 10.1080/07929978.2015.1006424

[B14] Cho R. J. Campbell M. J. (2000). Transcription, genomes, function. Trends Genet. 16, 409–415. 10.1016/S0168-9525(00)02065-5 10973070

[B15] Choi C. H. (2005). ABC transporters as multidrug resistance mechanisms and the development of chemosensitizers for their reversal. Cancer Cell Int. 5:30. 10.1186/1475-2867-5-3 16202168 PMC1277830

[B16] Clarke A. Desikan R. Hurst R. D. Hancock J. T. Neill S. J. (2000). NO way back: nitric oxide and programmed cell death in *Arabidopsis thaliana* suspension cultures. Plant J. 24, 667–677. 10.1046/j.1365-313x.2000.00911.x 11123805

[B17] Clay N. K. Adio A. M. Denoux C. Jander G. Ausubel F. M. (2009). Glucosinolate metabolites required for an Arabidopsis innate immune response. Science 323, 95–101. 10.1126/science.1164627 19095898 PMC2630859

[B18] Corpas F. J. Barroso J. B. Carreras A. Valderrama R. Palma J. M. León A. M.. (2006). Constitutive arginine-dependent nitric oxide synthase activity in different organs of pea seedlings during plant development. Planta 224, 246–254. 10.1007/s00425-005-0205-9 16397797

[B19] Corpas F. J. Palma J. M. del Río L. A. Barroso J. B. (2009). Evidence supporting the existence of L-arginine-dependent nitric oxide synthase activity in plants. New Phytol. 184, 9–14. 10.1111/j.1469-8137.2009.02989.x 19659743

[B20] Culotta E. Koshland D. E. (1992). NO news is good news. Science 258, 1862–1865. 10.1126/science.1361684 1361684

[B21] de Abreu-Neto J. B. Turchetto-Zolet A. C. de Oliveira L. F. Zanettini M. H. Margis-Pinheiro M. (2013). Heavy metal-associated isoprenylated plant protein (HIPP): characterization of a family of proteins exclusive to plants. FEBS J. 280, 1604–1616. 10.1111/febs.12159 23368984

[B22] Delledonne M. Xia Y. J. Dixon R. A. Lamb C. (1998). Nitric oxide functions as a signal in plant disease resistance. Nature 394, 585–588. 10.1038/29087 9707120

[B23] Delledonne M. Zeier J. Marocco A. Lamb C. (2001). Signal interactions between nitric oxide and reactive oxygen intermediates in the plant hypersensitive disease resistance response. Proc. Natl. Acad. Sci. U.S.A. 98, 13454–13459. 10.1073/pnas.231178298 11606758 PMC60892

[B24] Desikan R. Mackerness S. A. H. Hancock J. T. Neill S. J. (2001). Regulation of the Arabidopsis transcriptome by oxidative stress. Plant Physiol. 127, 159–172. 10.1104/pp.127.1.159 11553744 PMC117972

[B25] Ding Y. Z. Shaholli D. Mou Z. L. (2015). A large-scale genetic screen for mutants with altered salicylic acid accumulation in Arabidopsis. Front. Plant Sci. 5:763. 10.3389/fpls.2014.00763 25610446 PMC4285869

[B26] Ditt R. F. Kerr K. F. de Figueiredo P. Delrow J. Comai L. Nester E. W. (2006). The *Arabidopsis thaliana* transcriptome in response to *Agrobacterium tumefaciens*. Mol. Plant Microbe Interact. 19, 665–681. 10.1094/MPMI-19-0665 16776300

[B27] Du Y. Y. Wang P. C. Chen J. Song C. P. (2008). Comprehensive functional analysis of the catalase gene family in *Arabidopsis thaliana*. J. Integr. Plant Biol. 50, 1318–1326. 10.1111/j.1744-7909.2008.00741.x 19017119

[B28] Dubovskaya L. V. Kolesneva E. V. Knyazev D. M. Volotovskii I. D. (2007). Protective role of nitric oxide during hydrogen peroxide-induced oxidative stress in tobacco plants. Russ. J. Plant Physiol. 54, 755–762. 10.1134/S1021443707060064

[B29] Durner J. Wendehenne D. Klessig D. F. (1998). Defense gene induction in tobacco by nitric oxide, cyclic GMP, and cyclic ADP-ribose. Proc. Natl. Acad. Sci. U.S.A. 95, 10328–10333. 10.1073/pnas.95.17.10328 9707647 PMC21508

[B30] Ehlting J. Chowrira S. G. Mattheus N. Aeschliman D. S. Arimura G. I. Bohlmann J. (2008). Comparative transcriptome analysis of *Arabidopsis thaliana* infested by diamond back moth (*Plutella xylostella*) larvae reveals signatures of stress response, secondary metabolism, and signalling. BMC Genomics 9:154. 10.1186/1471-2164-9-154 18400103 PMC2375910

[B31] Eulgem T. (2005). Regulation of the Arabidopsis defense transcriptome. Trends Plant Sci. 10, 71–78. 10.1016/j.tplants.2004.12.006 15708344

[B32] Feechan A. Kwon E. Yun B. W. Wang Y. Q. Pallas J. A. Loake G. J. (2005). A central role for S-nitrosothiols in plant disease resistance. Proc. Natl. Acad. Sci. U.S.A. 102, 8054–8059. 10.1073/pnas.0501456102 15911759 PMC1142375

[B33] Feng B. M. Lu D. H. Ma X. Peng Y. B. Sun Y. J. Ning G.. (2012). Regulation of the Arabidopsis anther transcriptome by DYT1 for pollen development. Plant J. 72, 612–624. 10.1111/j.1365-313X.2012.05104.x 22775442

[B34] Ferrarini A. De Stefano M. Baudouin E. Pucciariello C. Polverari A. Puppo A.. (2008). Expression of *Medicago truncatula* genes responsive to nitric oxide in pathogenic and symbiotic conditions. Mol. Plant Microbe Interact. 21, 781–790. 10.1094/MPMI-21-6-0781 18624641

[B35] Flicek P. Amode M. R. Barrell D. Beal K. Billis K. Brent S.. (2014). Ensembl 2014. Nucleic Acids Res. 42, D749–D755. 10.1093/nar/gkt1196 24316576 PMC3964975

[B36] Foissner I. Wendehenne D. Langebartels C. Durner J. (2000). *In vivo* imaging of an elicitor-induced nitric oxide burst in tobacco. Plant J. 23, 817–824. 10.1046/j.1365-313X.2000.00835.x 10998192

[B37] Foresi N. Correa-Aragunde N. Parisi G. Caló G. Salerno G. Lamattina L. (2010). Characterization of a nitric oxide synthase from the plant kingdom: no generation from the green alga *Ostreococcus tauri* is light irradiance and growth phase dependent. Plant Cell 22, 3816–3830. 10.1105/tpc.109.073510 21119059 PMC3015112

[B38] Fourcroy P. Vansuyt G. Kushnir S. Inzé D. Briat J. F. (2004). Iron-regulated expression of a cytosolic ascorbate peroxidase encoded by the *APX1* gene in Arabidopsis seedlings. Plant Physiol. 134, 605–613. 10.1104/pp.103.029876 14739345 PMC344537

[B39] Foyer C. H. Noctor G. (2005). Redox homeostasis and antioxidant signaling: a metabolic interface between stress perception and physiological responses. Plant Cell. 17, 1866–1875. 10.1105/tpc.105.033589 15987996 PMC1167537

[B40] Freschi L. (2013). Nitric oxide and phytohormone interactions: current status and perspectives. Front. Plant Sci. 4:398. 10.3389/fpls.2013.00398 24130567 PMC3793198

[B41] Galatro A. Puntarulo S. Guiamet J. J. Simontacchi M. (2013). Chloroplast functionality has a positive effect on nitric oxide level in soybean cotyledons. Plant Physiol Biochem. 66, 26–33. 10.1016/j.plaphy.2013.01.019 23466744

[B42] Gandotra N. Coughlan S. J. Nelson T. (2013). The Arabidopsis leaf provascular cell transcriptome is enriched in genes with roles in vein patterning. Plant J. 74, 48–58. 10.1111/tpj.12100 23437797

[B43] García-Sánchez S. Bernales I. Cristobal S. (2015). Early response to nanoparticles in the Arabidopsis transcriptome compromises plant defence and root-hair development through salicylic acid signalling. BMC Genomics 16:341. 10.1186/s12864-015-1530-4 25903678 PMC4417227

[B44] Gierth M. Mäser P. (2007). Potassium transporters in plants - Involvement in K+ acquisition, redistribution and homeostasis. FEBS Lett. 581, 2348–2356. 10.1016/j.febslet.2007.03.035 17397836

[B45] Glass A. D. M. Britto D. T. Kaiser B. N. Kinghorn J. R. Kronzucker H. J. Kumar A.. (2002). The regulation of nitrate and ammonium transport systems in plants. J. Exp. Bot. 53, 855–864. 10.1093/jexbot/53.370.855 11912228

[B46] Gould K. S. Lamotte O. Klinguer A. Pugin A. Wendehenne D. (2003). Nitric oxide production in tobacco leaf cells: a generalized stress response? Plant Cell Environ. 26, 1851–1862. 10.1046/j.1365-3040.2003.01101.x

[B47] Grennan A. K. (2007). An analysis of the Arabidopsis pollen transcriptome. Plant Physiol. 145, 3–4. 10.1104/pp.104.900237 17823270 PMC1976589

[B48] Grün S. Lindermayr C. Sell S. Durner J. (2006). Nitric oxide and gene regulation in plants. J. Exp. Bot. 57, 507–516. 10.1093/jxb/erj053 16396997

[B49] Guo F. Q. Okamoto M. Crawford N. M. (2003). Identification of a plant nitric oxide synthase gene involved in hormonal signaling. Science 302, 100–103. 10.1126/science.1086770 14526079

[B50] Gupta K. J. Zabalza A. van Dongen J. T. (2009). Regulation of respiration when the oxygen availability changes. Physiol. Plant 137, 383–391. 10.1111/j.1399-3054.2009.01253.x 19549068

[B51] Gusarov I. Shatalin K. Starodubtseva M. Nudler E. (2009). Endogenous nitric oxide protects bacteria against a wide spectrum of antibiotics. Science 325, 1380–1384. 10.1126/science.1175439 19745150 PMC2929644

[B52] He Y. K. Tang R. H. Hao Y. Stevens R. D. Cook C. W. Am S. M.. (2004). Nitric oxide represses the Arabidopsis floral transition. Science 305, 1968–1971. 10.1126/science.1098837 15448272

[B53] Hill K. (2015). Post-translational modifications of hormone-responsive transcription factors: the next level of regulation. J. Exp. Bot. 66, 4933–4945. 10.1093/jxb/erv273 26041319

[B54] Hirschi K. D. (2004). The calcium conundrum. Both versatile nutrient and specific signal. Plant Physiol. 136, 2438–2442. 10.1104/pp.104.046490 15375199 PMC523310

[B55] Hoff T. Stummann B. M. Henningsen K. W. (1992). Structure, function and regulation of nitrate reductase in higher plants. Physiol. Plant 84, 616–624. 10.1111/j.1399-3054.1992.tb04712.x

[B56] Huang X. Stettmaier K. Michel C. Hutzler P. Mueller M. J. Durner J. (2004). Nitric oxide is induced by wounding and influences jasmonic acid signaling in Arabidopsis thaliana. Planta 218, 938–946. 10.1007/s00425-003-1178-1 14716563

[B57] Hu J. L. Huang X. H. Chen L. C. Sun X. W. Lu C. M. Zhang L. X.. (2015). Site-specific nitrosoproteomic identification of endogenously S-nitrosylated proteins in Arabidopsis. Plant Physiol. 167, 1731–1746. 10.1104/pp.15.00026 25699590 PMC4378176

[B58] Jasid S. Simontacchi M. Bartoli C. G. Puntarulo S. (2006). Chloroplasts as a nitric oxide cellular source. Effect of reactive nitrogen species on chloroplastic lipids and proteins. Plant Physiol. 142, 1246–1255. 10.1104/pp.106.086918 16980561 PMC1630751

[B59] Jones M. A. Raymond M. J. Smirnoff N. (2006). Analysis of the root-hair morphogenesis transcriptome reveals the molecular identity of six genes with roles in root-hair development in Arabidopsis. Plant J. 45, 83–100. 10.1111/j.1365-313X.2005.02609.x 16367956

[B60] Kang H. G. Kim J. Kim B. Jeong H. Choi S. H. Kim E. K.. (2011). Overexpression of FTL1/DDF1, an AP2 transcription factor, enhances tolerance to cold, drought, and heat stresses in *Arabidopsis thaliana*. Plant Sci. 180, 634–641. 10.1016/j.plantsci.2011.01.002 21421412

[B61] Kawakita K. Shahjahan M. M. Takemoto D. (2010). Plant defense-related activities of NO producing elicitor candidates on potato and *Nicotiana benthamiana*. Nitric Oxide 22, S77.

[B62] Kawakita K. Yamamoto A. Katou S. Yoshioka H. Doke N. (2004). Involvement of NO in plant defense responses and role of nitrate reductase in NO production. Nitric Oxide 11, 37.

[B63] Kempema L. A. Cui X. P. Holzer F. M. Walling L. L. (2007). Arabidopsis transcriptome changes in response to phloem-feeding silverleaf whitefly nymphs. Similarities and distinctions in responses to aphids. Plant Physiol. 143, 849–865. 10.1104/pp.106.090662 17189325 PMC1803730

[B64] Kneeshaw S. Gelineau S. Tada Y. Loake G. J. Spoel S. H. (2014). Selective protein denitrosylation activity of thioredoxin-h5 modulates plant immunity. Mol. Cell 56, 153–162. 10.1016/j.molcel.2014.08.003 25201412

[B65] Kumar D. Klessig D. F. (2000). Differential induction of tobacco MAP kinases by the defense signals nitric oxide, salicylic acid, ethylene, and jasmonic acid. Mol. Plant Microbe Interact. 13, 347–351. 10.1094/MPMI.2000.13.3.347 10707361

[B66] Kwon E. Feechan A. Yun B. W. Hwang B. H. Pallas J. A. Kang J. G.. (2012). AtGSNOR1 function is required for multiple developmental programs in Arabidopsis. Planta 236, 887–900. 10.1007/s00425-012-1697-8 22767201

[B67] Lam Y. W. Yuan Y. Isaac J. Babu C. V. S. Meller J. Ho S. M. (2010). Comprehensive identification and modified-site mapping of S-nitrosylated targets in prostate epithelial cells. PLoS ONE 5:e9075. 10.1371/journal.pone.0009075 20140087 PMC2816712

[B68] Lee U. Wie C. Fernandez B. O. Feelisch M. Vierling E. (2008). Modulation of nitrosative stress by S-nitrosoglutathione reductase is critical for thermotolerance and plant growth in Arabidopsis. Plant Cell 20, 786–802. 10.1105/tpc.107.052647 18326829 PMC2329944

[B69] Li J. Liu J. T. Wang G. Q. Cha J. Y. Li G. N. Chen S.. (2015). A chaperone function of *NO CATALASE ACTIVITY1* is required to maintain catalase activity and for multiple stress responses in Arabidopsis. Plant Cell 27, 908–925. 10.1105/tpc.114.135095 25700484 PMC4558663

[B70] Li X. N. Jiang H. D. Liu F. L. Cai J. Dai T. B. Cao W. X. (2013). Induction of chilling tolerance in wheat during germination by pre-soaking seed with nitric oxide and gibberellin. Plant Growth Regul. 71, 31–40. 10.1007/s10725-013-9805-8

[B71] Li X. Shen Z. Airoian R. V. Briggs S. P. (2007). A global view of transcriptome and proteome in compatible Arabidopsis-root knot nematode interaction. J. Nematol. 39, 73–74.

[B72] Liu P. P. Yang Y. Pichersky E. Klessig D. F. (2010). Altering expression of Benzoic Acid/Salicylic Acid Carboxyl Methyltransferase 1 compromises systemic acquired resistance and PAMP-triggered immunity in Arabidopsis. Mol. Plant Microbe Interact. 23, 82–90. 10.1094/MPMI-23-1-0082 19958141

[B73] Lopez-Molina L. Mongrand B. McLachlin D. T. Chait B. T. Chua N. H. (2002). ABI5 acts downstream of ABI3 to execute an ABA-dependent growth arrest during germination. Plant J. 32, 317–328. 10.1046/j.1365-313X.2002.01430.x 12410810

[B74] Lu T. T. Lu G. J. Fan D. L. Zhu C. R. Li W. Zhao Q. A.. (2010). Function annotation of the rice transcriptome at single-nucleotide resolution by RNA-seq. Genome Res. 20, 1238–1249. 10.1101/gr.106120.110 20627892 PMC2928502

[B75] Maag D. Erb M. Köllner T. G. Gershenzon J. (2015). Defensive weapons and defense signals in plants: some metabolites serve both roles. BioEssays 37, 167–174. 10.1002/bies.201400124 25389065

[B76] Magome H. Yamaguchi S. Hanada A. Kamiya Y. Oda K. (2008). The DDF1 transcriptional activator upregulates expression of a gibberellin-deactivating gene, *GA2ox7*, under high-salinity stress in Arabidopsis. Plant J. 56, 613–626. 10.1111/j.1365-313X.2008.03627.x 18643985

[B77] Malik S. I. Hussain A. Yun B. W. Spoel S. H. Loake G. J. (2011). GSNOR-mediated de-nitrosylation in the plant defence response. Plant Sci. 181, 540–544. 10.1016/j.plantsci.2011.04.004 21893250

[B78] Martínez-Ruiz A. Lamas S. (2004). Detection and proteomic identification of S-nitrosylated proteins in endothelial cells. Arch. Biochem. Biophys. 423, 192–199. 10.1016/j.abb.2003.12.006 14871481

[B79] Matsui A. Ishida J. Morosawa T. Mochizuki Y. Kaminuma E. Endo T. A.. (2008). Arabidopsis transcriptome analysis under drought, cold, high-salinity and ABA treatment conditions using a tiling array. Plant Cell Physiol. 49, 1135–1149. 10.1093/pcp/pcn101 18625610

[B80] Matsumoto A. Comatas K. E. Liu L. M. Stamler J. S. (2003). Screening for nitric oxide-dependent protein-protein interactions. Science 301, 657–661. 10.1126/science.1079319 12893946

[B81] Matsuura H. Yamamoto Y. Muraoka M. Akaishi K. Hori Y. Uemura K.. (2013). Development of surface-engineered yeast cells displaying phytochelatin synthase and their application to cadmium biosensors by the combined use of pyrene-excimer fluorescence. Biotechnol. Prog. 29, 1197–1202. 10.1002/btpr.1789 23926095

[B82] Mhamdi A. Queval G. Chaouch S. Vanderauwera S. Van Breusegem F. Noctor G. (2010). Catalase function in plants: a focus on Arabidopsis mutants as stress-mimic models. J. Exp. Bot. 61, 4197–4220. 10.1093/jxb/erq282 20876333

[B83] Misra A. N. Vladkova R. Singh R. Misra M. Dobrikova A. G. Apostolova E. L. (2014). Action and target sites of nitric oxide in chloroplasts. Nitric Oxide 39, 35–45. 10.1016/j.niox.2014.04.003 24731839

[B84] Moncada S. Palmer R. M. J. (1993). Cellular Nitric-Oxide Synthesis - a Citation-Classic commentary on nitric oxide release accounts for the biological activity of endothelium-derived relaxing factor by Palmer, R. M. J., Ferrige, A. G., and Moncada, S. Cc Life Sci. 8. 10.1038/327524a0 3495737

[B85] Mur L. A. J. Prats E. Pierre S. Hall M. A. Hebelstrup K. H. (2013). Integrating nitric oxide into salicylic acid and jasmonic acid/ethylene plant defense pathways. Front. Plant Sci. 4:215. 10.3389/fpls.2013.00215 23818890 PMC3694216

[B86] Nanao M. H. Vinos-Poyo T. Brunoud G. Thévenon E. Mazzoleni M. Mast D.. (2014). Structural basis for oligomerization of auxin transcriptional regulators. Nat Commun. 5:3617. 10.1038/ncomms4617 24710426

[B87] Nott A. Watson P. M. Robinson J. D. Crepaldi L. Riccio A. (2008). S-nitrosylation of histone deacetylase 2 induces chromatin remodelling in neurons. Nature 455, U411–U467. 10.1038/nature07238 18754010

[B88] Okada A. Ura S. Matsuda A. Ito T. Fukasaku N. Shiraishi N. (2004). Molecular mechanism of nitric oxide production by nitrate reductase in higher plants. Nitric Oxide 11, 88.

[B89] Palmieri M. C. Sell S. Huang X. Scherf M. Werner T. Durner J.. (2008). Nitric oxide-responsive genes and promoters in *Arabidopsis thaliana*: a bioinformatics approach. J. Exp. Bot. 59, 177–186. 10.1093/jxb/erm345 18272923

[B90] Paponov I. A. Paponov M. Teale W. Menges M. Chakrabortee S. Murray J. A. H.. (2008). Comprehensive transcriptome analysis of auxin responses in Arabidopsis. Mol. Plant 1, 321–337. 10.1093/mp/ssm021 19825543

[B91] Parani M. Rudrabhatla S. Myers R. Weirich H. Smith B. Leaman D. W.. (2004). Microarray analysis of nitric oxide responsive transcripts in Arabidopsis. Plant Biotechnol. J. 2, 359–366. 10.1111/j.1467-7652.2004.00085.x 17134397

[B92] París R. Iglesias M. J. Terrile M. C. Casalongué C. A. (2013). Functions of S-nitrosylation in plant hormone networks. Front. Plant Sci. 4:294. 10.3389/fpls.2013.00294 23914202 PMC3729995

[B93] Patel R. K. Jain M. (2012). NGS QC Toolkit: A toolkit for quality control of next generation sequencing data. PLoS ONE 7:e30619. 10.1371/journal.pone.0030619 22312429 PMC3270013

[B94] Pei Z. M. Baizabal-Aguirre V. M. Allen G. J. Schroeder J. I. (1998). A transient outward-rectifying K^+^ channel current down-regulated by cytosolic Ca^2+^ in *Arabidopsis thaliana* guard cells. Proc. Natl. Acad. Sci. U.S.A. 95, 6548–6553. 10.1073/pnas.95.11.6548 9601004 PMC27872

[B95] Peng M. S. Bi Y. M. Zhu T. Rothstein S. J. (2007). Genome-wide analysis of Arabidopsis responsive transcriptome to nitrogen limitation and its regulation by the ubiquitin ligase gene NLA. Plant Mol. Biol. 65, 775–797. 10.1007/s11103-007-9241-0 17885809

[B96] Petrouleas V. Diner B. A. (1990). Formation by NO of nitrosyl adducts of redox components of the photosystem II reaction center. I. NO binds to the acceptor-side non-heme iron. Biochim. Biophys. Acta 1015, 131–140.

[B97] Polverari A. Molesini B. Pezzotti M. Buonaurio R. Marte M. Delledonne M. (2003). Nitric oxide-mediated transcriptional changes in *Arabidopsis thaliana*. Mol. Plant Microbe Interact. 16, 1094–1105. 10.1094/MPMI.2003.16.12.1094 14651343

[B98] Postnikova O. A. Nemchinov L. G. (2012). Comparative analysis of microarray data in Arabidopsis transcriptome during compatible interactions with plant viruses. Virol J. 9:101. 10.1186/1743-422X-9-101 22643110 PMC3430556

[B99] Rae L. Lao N. T. Kavanagh T. A. (2011). Regulation of multiple aquaporin genes in Arabidopsis by a pair of recently duplicated DREB transcription factors. Planta. 234, 429–444. 10.1007/s00425-011-1414-z 21509693

[B100] Rodríguez-Navarro A. Rubio F. (2006). High-affinity potassium and sodium transport systems in plants. J. Exp. Bot. 57, 1149–1160. 10.1093/jxb/erj068 16449373

[B101] Saeed A. I. Sharov V. White J. Li J. Liang W. Bhagabati N.. (2003). TM4: A free, open-source system for microarray data management and analysis. BioTechniques 34, 374–378. 12613259 10.2144/03342mt01

[B102] Sang J. R. Jiang M. Y. Lin F. Xu S. C. Zhang A. Tan M. P. (2008a). Nitric oxide reduces hydrogen peroxide accumulation involved in water stress-induced subcellular anti-oxidant defense in maize plants. J. Integr. Plant Biol. 50, 231–243. 10.1111/j.1744-7909.2007.00594.x 18713446

[B103] Sang J. R. Zhang A. Lin F. Tan M. P. Jiang M. Y. (2008b). Cross-talk between calcium-calmodulin and nitric oxide in abscisic acid signaling in leaves of maize plants. Cell Res. 18, 577–588. 10.1038/cr.2008.39 18364679

[B104] Sanz L. Albertos P. Mateos I. Sánchez-Vicente I. Lechón T. Fernández-Marcos M.. (2015). Nitric oxide (NO) and phytohormones crosstalk during early plant development. J. Exp. Bot. 66, 2857–2868. 10.1093/jxb/erv213 25954048

[B105] Shi K. Li X. Zhang H. Zhang G. Q. Liu Y. R. Zhou Y. H.. (2015). Guard cell hydrogen peroxide and nitric oxide mediate elevated CO2-induced stomatal movement in tomato. New Phytol. 208, 342–353. 10.1111/nph.13621 26308648

[B106] Storozhenko S. De Pauw P. Van Montagu M. Inzé D. Kushnir S. (1998). The heat-shock element is a functional component of the Arabidopsis *APX1* gene promoter. Plant Physiol. 118, 1005–1014. 10.1104/pp.118.3.1005 9808745 PMC34773

[B107] Sun X. L. Gilroy E. M. Chini A. Nurmberg P. L. Hein I. Lacomme C.. (2011). *ADS1* encodes a MATE-transporter that negatively regulates plant disease resistance. New Phytol. 192, 471–482. 10.1111/j.1469-8137.2011.03820.x 21762165

[B108] Tada Y. Spoel S. H. Pajerowska-Mukhtar K. Mou Z. L. Song J. Q. Wang C. (2008). Plant immunity requires conformational charges of NPR1 via S-nitrosylation and thioredoxins. Science 321, 952–956. 10.1126/science.1156970 18635760 PMC3833675

[B109] Tang S. Liang H. Y. Yan D. H. Zhao Y. Han X. Carlson J. E.. (2013). *Populus euphratica*: the transcriptomic response to drought stress. Plant Mol. Biol. 83, 539–557. 10.1007/s11103-013-0107-3 23857471

[B110] Tanou G. Job C. Belghazi M. Molassiotis A. Diamantidis G. Job D. (2010). Proteomic signatures uncover hydrogen peroxide and nitric oxide cross-talk signaling network in citrus plants. J Proteome Res. 9, 5994–6006. 10.1021/pr100782h 20825250

[B111] Tanou G. Job C. Rajjou L. Arc E. Belghazi M. Diamantidis G.. (2009). Proteomics reveals the overlapping roles of hydrogen peroxide and nitric oxide in the acclimation of citrus plants to salinity. Plant J. 60, 795–804. 10.1111/j.1365-313X.2009.04000.x 19682288

[B112] Tavares C. P. Vernal J. Delena R. A. Lamattina L. Cassia R. Terenzi H. (2014). S-nitrosylation influences the structure and DNA binding activity of AtMYB30 transcription factor from *Arabidopsis thaliana*. Biochim. Biophys. Acta Proteins Proteom. 1844, 810–817. 10.1016/j.bbapap.2014.02.015 24583075

[B113] Terrile M. C. París R. Calderón-Villalobos L. I. A. Iglesias M. J. Lamattina L. Estelle M.. (2012). Nitric oxide influences auxin signaling through S-nitrosylation of the Arabidopsis TRANSPORT INHIBITOR RESPONSE 1 auxin receptor. Plant J. 70, 492–500. 10.1111/j.1365-313X.2011.04885.x 22171938 PMC3324642

[B114] Tewari R. K. Prommer J. Watanabe M. (2013). Endogenous nitric oxide generation in protoplast chloroplasts. Plant Cell Rep. 32, 31–44. 10.1007/s00299-012-1338-5 22971939

[B115] Thimm O. Bläsing O. Gibon Y. Nagel A. Meyer S. Krüger P.. (2004). MAPMAN: a user-driven tool to display genomics data sets onto diagrams of metabolic pathways and other biological processes. Plant J. 37, 914–939. 10.1111/j.1365-313X.2004.02016.x 14996223

[B116] Trapnell C. Pachter L. Salzberg S. L. (2009). TopHat: discovering splice junctions with RNA-Seq. Bioinformatics 25, 1105–1111. 10.1093/bioinformatics/btp120 19289445 PMC2672628

[B117] Trapnell C. Williams B. A. Pertea G. Mortazavi A. Kwan G. van Baren M. J.. (2010). Transcript assembly and quantification by RNA-Seq reveals unannotated transcripts and isoform switching during cell differentiation. Nat. Biotechnol. 28, 511–515. 10.1038/nbt.1621 20436464 PMC3146043

[B118] Umbrasaite J. Schweighofer A. Meskiene I. (2011). Substrate analysis of Arabidopsis PP2C-type protein phosphatases. Methods Mol. Biol. 779, 149–161. 10.1007/978-1-61779-264-9_8 21837565

[B119] Urbanczyk-Wochniak E. Usadel B. Thimm O. Nunes-Nesi A. Carrari F. Davy M.. (2006). Conversion of MapMan to allow the analysis of transcript data from Solanaceous species: effects of genetic and environmental alterations in energy metabolism in the leaf. Plant Mol. Biol. 60, 773–792. 10.1007/s11103-005-5772-4 16649112

[B120] Usadel B. Nagel A. Thimm O. Redestig H. Blaesing O. E. Palacios-Rojas N.. (2005). Extension of the visualization tool MapMan to allow statistical analysis of arrays, display of coresponding genes, and comparison with known responses. Plant Physiol. 138, 1195–1204. 10.1104/pp.105.060459 16009995 PMC1176394

[B121] Wang L. Guo Y. J. Jia L. X. Chu H. Y. Zhou S. Chen K. M.. (2014). Hydrogen peroxide acts upstream of nitric oxide in the heat shock pathway in Arabidopsis seedlings. Plant Physiol. 164, 2184–2196. 10.1104/pp.113.229369 24510762 PMC3982771

[B122] Wang P. Du Y. Li Y. Ren D. Song C. P. (2010). Hydrogen peroxide-mediated activation of MAP kinase 6 modulates nitric oxide biosynthesis and signal transduction in Arabidopsis. Plant Cell 22, 2981–2998. 10.1105/tpc.109.072959 20870959 PMC2965546

[B123] Wang R. S. Pandey S. Li S. Gookin T. E. Zhao Z. X. Albert R.. (2011). Common and unique elements of the ABA-regulated transcriptome of Arabidopsis guard cells. BMC Genomics. 12:216. 10.1186/1471-2164-12-216 21554708 PMC3115880

[B124] Wang Y. Q. Feechan A. Yun B. W. Shafiei R. Hofmann A. Taylor P.. (2009). S-Nitrosylation of AtSABP3 antagonizes the expression of plant immunity. J. Biol. Chem. 284, 2131–2137. 10.1074/jbc.M806782200 19017644

[B125] Wang Y. Z. Moreau M. Baek S. H. Dzikovski B. Shapleigh J. P. Crane B. R. (2006). Assessing the involvement of NO and AtNOS1 in plant defense using GFP-reporter system and NO spin trapping. Nitric Oxide 14, A2 10.1016/j.niox.2006.04.010

[B126] Weber M. Trampczynska A. Clemens S. (2006). Comparative transcriptome analysis of toxic metal responses in *Arabidopsis thaliana* and the Cd2^+^-hypertolerant facultative metallophyte *Arabidopsis halleri*. Plant Cell Environ. 29, 950–963. 10.1111/j.1365-3040.2005.01479.x 17087478

[B127] Weeda S. Zhang N. Zhao X. L. Ndip G. Guo Y. D. Buck G. A.. (2014). Arabidopsis transcriptome analysis reveals key roles of melatonin in plant defense systems. PLoS ONE 9:e93462. 10.1371/journal.pone.0093462 24682084 PMC3969325

[B128] Wildermuth M. C. Dewdney J. Wu G. Ausubel F. M. (2001). Isochorismate synthase is required to synthesize salicylic acid for plant defence. Nature 414, 562–565. 10.1038/35107108 11734859

[B129] Wilson I. D. Neill S. J. Hancock J. T. (2008). Nitric oxide synthesis and signalling in plants. Plant Cell Environ. 31, 622–631. 10.1111/j.1365-3040.2007.01761.x 18034772

[B130] Woo J. MacPherson C. R. Liu J. Wang H. Kiba T. Hannah M. A.. (2012). The response and recovery of the *Arabidopsis thaliana* transcriptome to phosphate starvation. BMC Plant Biol. 12:62. 10.1186/1471-2229-12-62 22553952 PMC3520718

[B131] Wu A. P. Gong L. Chen X. Wang J. X. (2014). Interactions between nitric oxide, gibberellic acid, and phosphorus regulate primary root growth in Arabidopsis. Biol Plantarum 58, 335–340. 10.1007/s10535-014-0408-7

[B132] Wu D. Chu H. Y. Jia L. X. Chen K. M. Zhao L. Q. (2015). A feedback inhibition between nitric oxide and hydrogen peroxide in the heat shock pathway in Arabidopsis seedlings. Plant Growth Regul. 75, 503–509. 10.1007/s10725-014-0014-x

[B133] Xiao S. Y. Charoenwattana P. Holcombe L. Turner J. G. (2003). The Arabidopsis genes RPW8.1 and RPW8.2 confer induced resistance to powdery mildew diseases in tobacco. Mol. Plant Microbe Interact. 16, 289–294. 10.1094/MPMI.2003.16.4.289 12744457

[B134] Xu W. Zhang S. S. Wang D. L. Liu J. Z. (2015). Drought tolerance of *nitric oxide associated 1* mutant of Arabidopsis is mostly due to its reduced transpiration as a result of smaller stature. Acta Physiol. Plant. 37:134 10.1007/s11738-015-1892-x

[B135] Yamasaki H. (2000). Nitrite-dependent nitric oxide production pathway: implications for involvement of active nitrogen species in photoinhibition *in vivo*. Philos. Trans. R. Soc. B 355, 1477–1488. 10.1098/rstb.2000.0708 11128001 PMC1692879

[B136] Yun B. W. Feechan A. Yin M. H. Saidi N. B. B. Le Bihan T. Yu M.. (2011). S-nitrosylation of NADPH oxidase regulates cell death in plant immunity. Nature 478, U264–U161. 10.1038/nature10427 21964330

[B137] Zanella L. Fattorini L. Brunetti P. Roccotiello E. Cornara L. D'Angeli S.. (2015). Overexpression of AtPCS1 in tobacco increases arsenic and arsenic plus cadmium accumulation and detoxification. Planta 243, 605–622. 10.1007/s00425-015-2428-8 26563149 PMC4757632

[B138] Zeeberg B. R. Feng W. M. Wang G. Wang M. D. Fojo A. T. Sunshine M.. (2003). GoMiner: a resource for biological interpretation of genomic and proteomic data. Genome Biol. 4:R28. 10.1186/gb-2003-4-4-r28 12702209 PMC154579

[B139] Zeng F. S. Sun F. K. Li L. L. Liu K. Zhan Y. G. (2014). Genome-scale transcriptome analysis in response to nitric oxide in birch cells: implications of the triterpene biosynthetic pathway. PLoS ONE 9:e116157. 10.1371/journal.pone.0116157 25551661 PMC4281108

[B140] Zhang B. Zheng L. P. Wang J. W. (2012). Nitric oxide elicitation for secondary metabolite production in cultured plant cells. Appl. Microbiol. Biotechnol. 93, 455–466. 10.1007/s00253-011-3658-8 22089384

[B141] Zhang H. W. Zhu H. F. Pan Y. J. Yu Y. X. Luan S. Li L. G. (2014). A DTX/MATE-type transporter facilitates abscisic acid efflux and modulates ABA sensitivity and drought tolerance in Arabidopsis. Mol. Plant 7, 1522–1532. 10.1093/mp/ssu063 24851876

[B142] Zhang L. S. Wang L. Yang Y. L. Cui J. Chang F. Wang Y. X.. (2015). Analysis of arabidopsis floral transcriptome: detection of new florally expressed genes and expansion of Brassicaceae-specific gene families. Front. Plant Sci. 5:802. 10.3389/fpls.2014.00802 25653662 PMC4299442

[B143] Zhang X. H. Shen L. Li F. J. Meng D. M. Sheng J. P. (2011). Methyl salicylate-induced arginine catabolism is associated with up-regulation of polyamine and nitric oxide levels and improves chilling tolerance in cherry tomato fruit. J. Agric. Food Chem. 59, 9351–9357. 10.1021/jf201812r 21790190

[B144] Zhao J. Yi H. L. (2014). Genome-wide transcriptome analysis of Arabidopsis response to sulfur dioxide fumigation. Mol. Genet. Genomics 289, 989–999. 10.1007/s00438-014-0870-0 24889700

[B145] Zhu X. F. Jiang T. Wang Z. W. Lei G. J. Shi Y. Z. Li G. X.. (2012). Gibberellic acid alleviates cadmium toxicity by reducing nitric oxide accumulation and expression of *IRT1* in *Arabidopsis thaliana*. J. Hazard. Mater. 239, 302–307. 10.1016/j.jhazmat.2012.08.077 23021314

[B146] Zou C. S. Yu D. Q. (2010). Analysis of the cold-responsive transcriptome in the mature pollen of Arabidopsis. J. Plant Biol. 53, 400–416. 10.1007/s12374-010-9129-4

