# Supplementary material for: Nitric Oxide Mediated Transcriptome Profiling Reveals Activation of Multiple Regulatory Pathways in Arabidopsis thaliana
Source: Front Plant Sci. 2016 Jun 29;7:975. doi: 10.3389/fpls.2016.00975 (PMC4926318; doi:10.3389/fpls.2016.00975)
Supplement: Supplementary file 9 [file Image1.PDF]

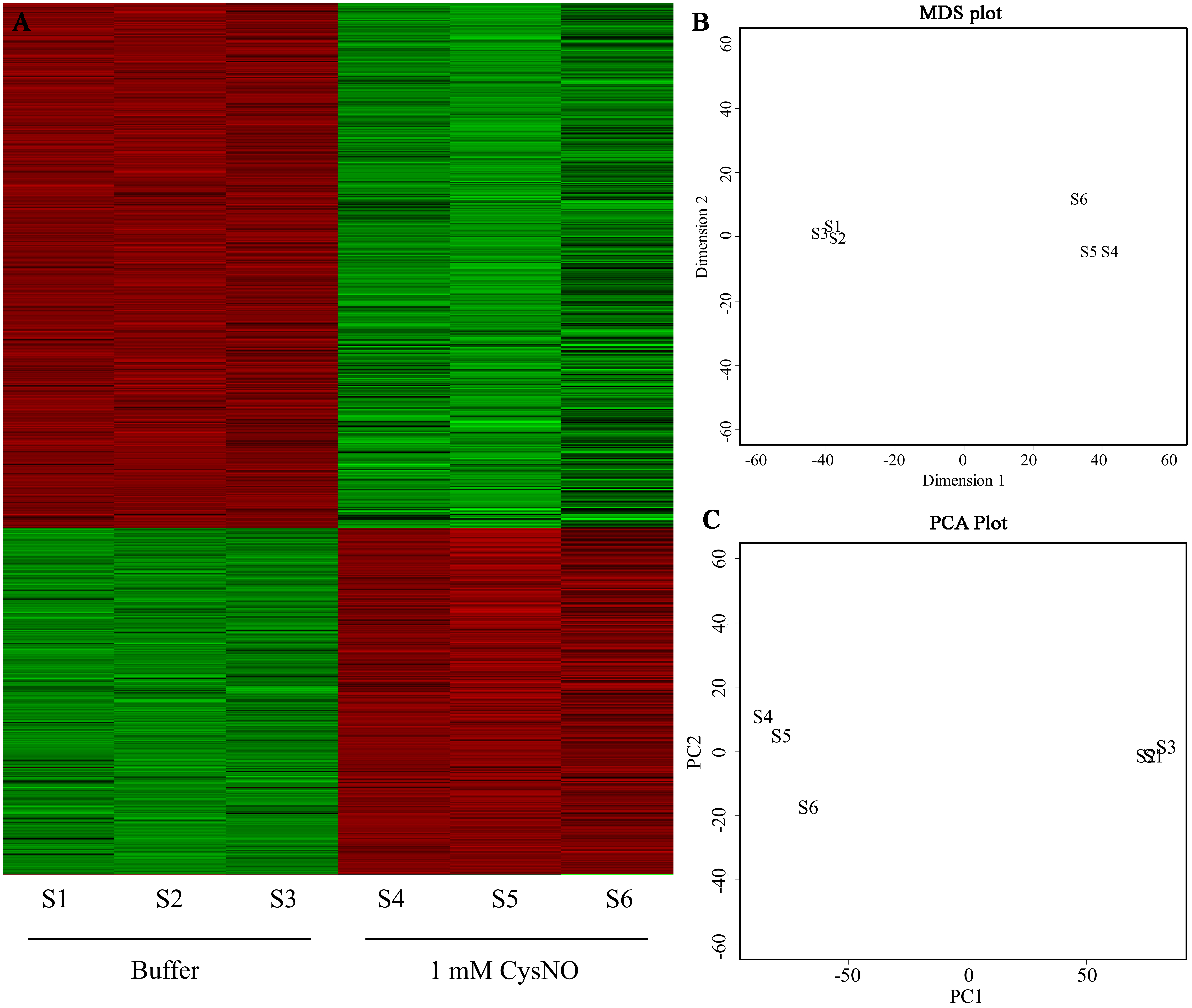

Supplementary Figure S1: Arabidopsis leaf transcriptome in response to 1 mM CysNO infiltration. (A). Heat map showing signal intensities of 1,165 differentially expressed genes (463 up-regulated and 702 down-regulated) in three replicates each of control (buffer-treated) and CysNO treated leaf samples. (B & C). Multi-dimensional scattered (MDS) and principal component analysis (PCA) plots showing average data dispersion of contorl and condition samples.
