# Supplementary material for: Nitric Oxide Mediated Transcriptome Profiling Reveals Activation of Multiple Regulatory Pathways in Arabidopsis thaliana
Source: Front Plant Sci. 2016 Jun 29;7:975. doi: 10.3389/fpls.2016.00975 (PMC4926318; doi:10.3389/fpls.2016.00975)
Supplement: Supplementary file 10 [file Image2.PDF]

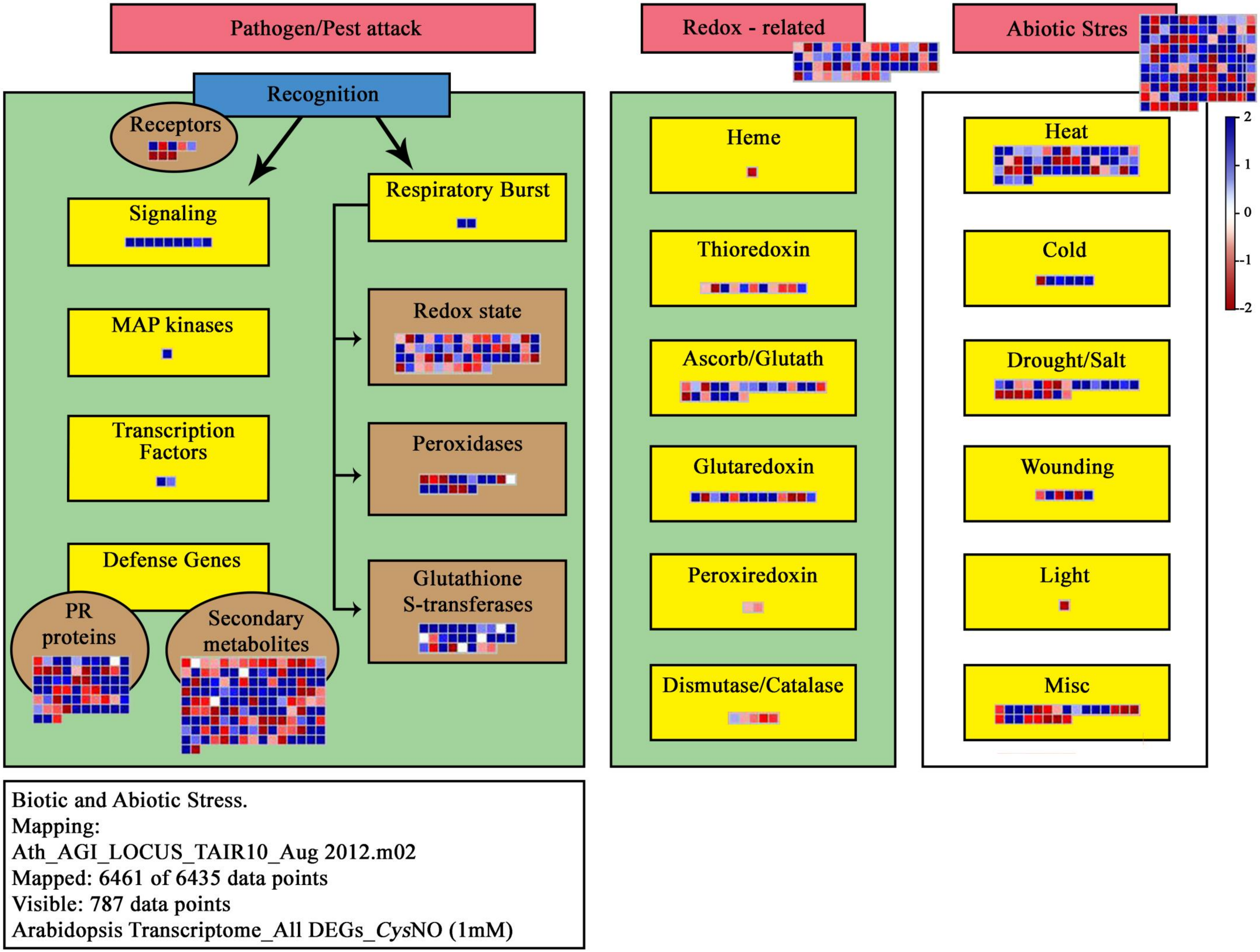

Supplementary figure S2. Differentially expressed Arabidopsis genes involved in biotic and abiotic stress. Differentially expressed genes (DEGs) in the RNA-Seq transcriptome of 1 mM CysNO-treated Arabidopsis leaves were analyzed using MapMan3.6.0. A total of 787 DEGs (319 down-regulated and 468 up-regulated) were found to be involved in biotic and abiotic stress responses and redox-related pathways. Down-regulated genes are represented by red squares while up-regulated genes are represented by blue squares. A detailed list of all these genes and their expression values can be found in Supplementary Table S3.
