# Supplementary material for: Nitric Oxide Mediated Transcriptome Profiling Reveals Activation of Multiple Regulatory Pathways in Arabidopsis thaliana
Source: Front Plant Sci. 2016 Jun 29;7:975. doi: 10.3389/fpls.2016.00975 (PMC4926318; doi:10.3389/fpls.2016.00975)
Supplement: Supplementary file 11 [file Image3.PDF]

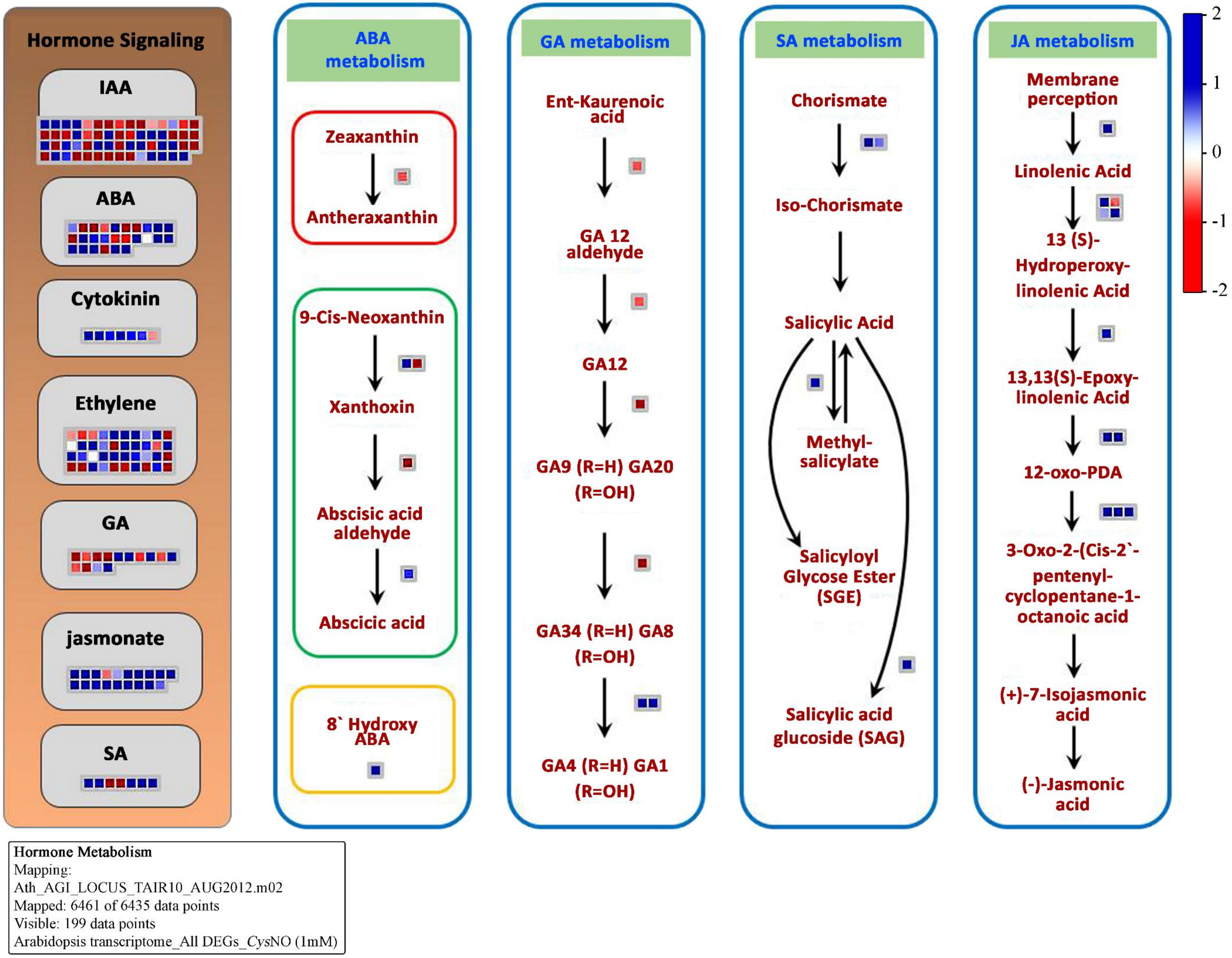

Supplementary figure S3: Differentially expressed Arabidopsis genes involved in hormone metabolism. Differentially expressed genes in the transcriptome of 1 mM CysNO-treated Arabidopsis leaves were analyzed using MapMan3.6.0. A total of 199 DEGs (75 down-regulated and 124 up-regulated) were found to be involved in the metabolism of various phytohormones. Down-regulated genes are represented by red squares whereas up-regulated genes are represented by blue squares. A detailed list of all these genes and their expression values can be found in Supplementary Table S4.
