# Supplementary material for: Nitric Oxide Mediated Transcriptome Profiling Reveals Activation of Multiple Regulatory Pathways in Arabidopsis thaliana
Source: Front Plant Sci. 2016 Jun 29;7:975. doi: 10.3389/fpls.2016.00975 (PMC4926318; doi:10.3389/fpls.2016.00975)
Supplement: Supplementary file 13 [file Image5.PDF]

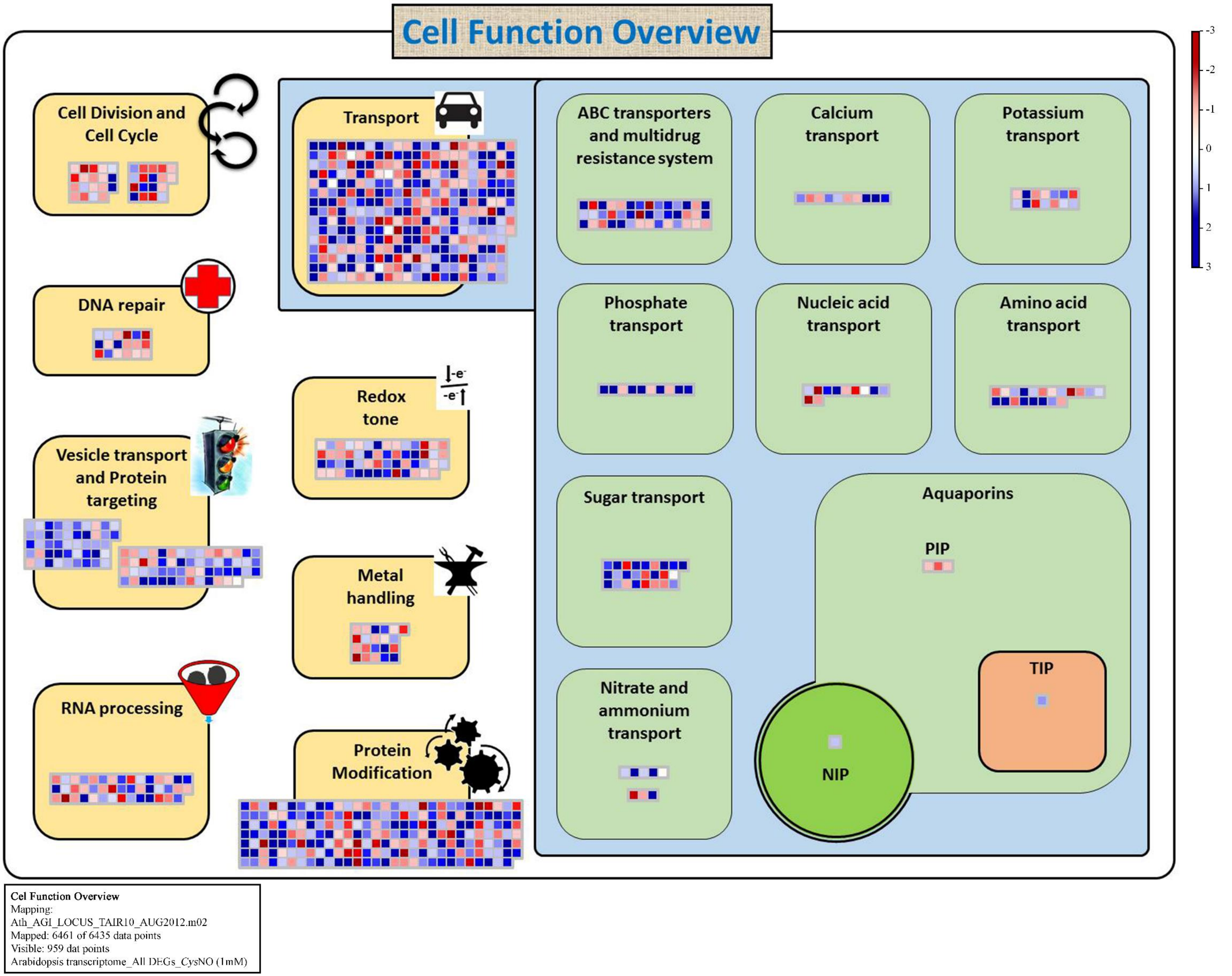

Supplementary Figure S5: Differentially expressed Arabidopsis genes involved in cellular functions. Differentially expressed genes in the transcriptome of 1mM *Cys*NO treated Arabidopsis leaves were analyzed using MapMan3.6.0. A total of 959 DEGs (372 down-regulated and 587 up-regulated) were found to be involved in various cellular functions mentioned. Down regulated genes are expressed by squares colored in red while up-regulated DEGs are represented by blue colored squares. Detailed list of all these genes and their expression values can be found in Supplementary Table S6.
