# Supplementary material for: Nitric Oxide Mediated Transcriptome Profiling Reveals Activation of Multiple Regulatory Pathways in Arabidopsis thaliana
Source: Front Plant Sci. 2016 Jun 29;7:975. doi: 10.3389/fpls.2016.00975 (PMC4926318; doi:10.3389/fpls.2016.00975)
Supplement: Supplementary file 14 [file Image6.PDF]

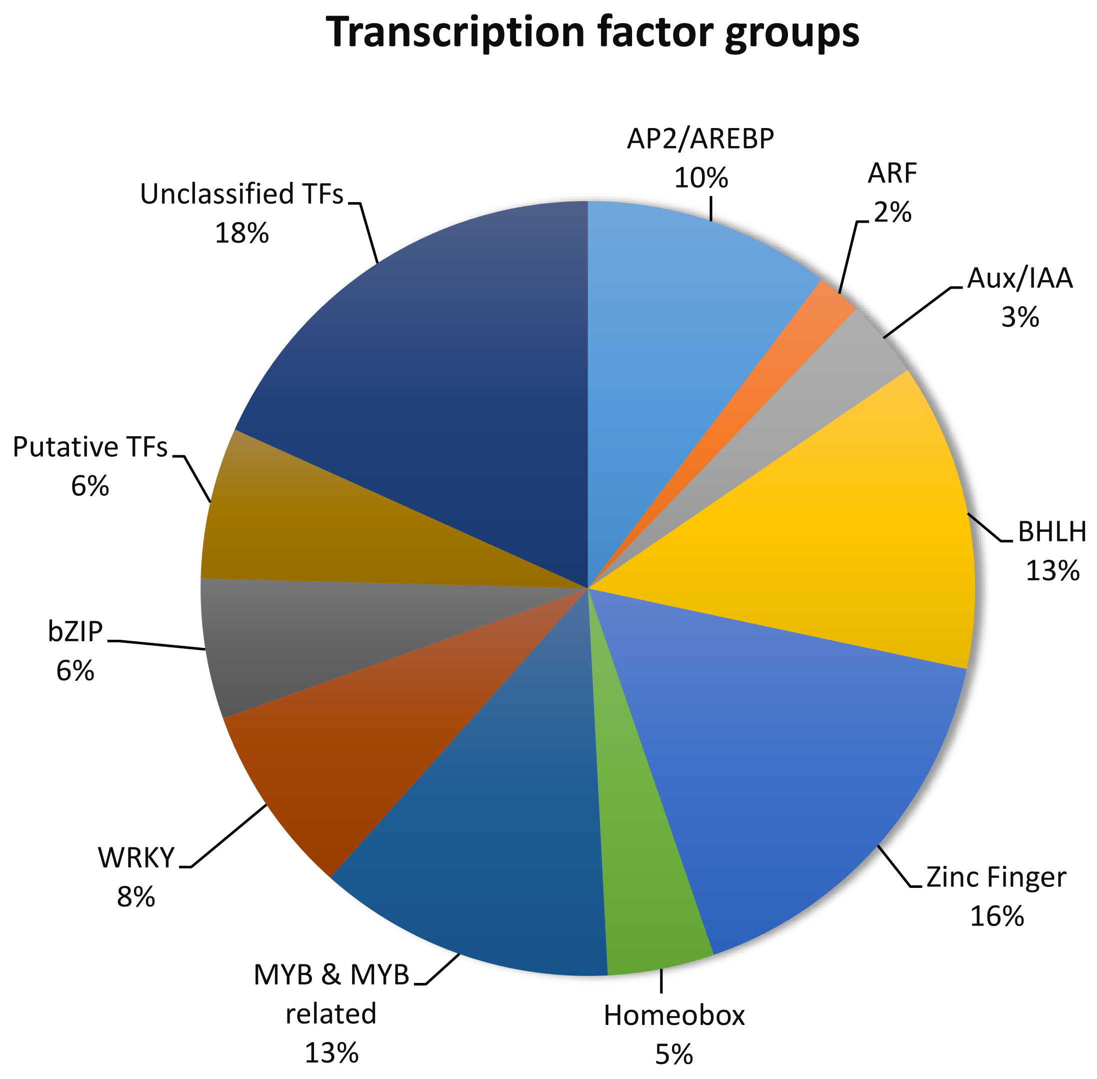

Supplementary Figure S6: Differentially expressed transcription factor groups in the Arabidopsis leaf transcriptome infiltrated with 1mM *Cys*NO. A total of 604 (274 down-regulated and 330 up-regulated) differentially expressed genes encoding known and putative transcription factors were found in the transcriptome of 1mM *Cys*NO treated Arabidopsis leaves. Detailed list of all these TFs and their expression values can be found in Supplementary Table S7.
