# Supplementary material for: Nitric Oxide Mediated Transcriptome Profiling Reveals Activation of Multiple Regulatory Pathways in Arabidopsis thaliana
Source: Front Plant Sci. 2016 Jun 29;7:975. doi: 10.3389/fpls.2016.00975 (PMC4926318; doi:10.3389/fpls.2016.00975)
Supplement: Supplementary file 15 [file Image7.PDF]

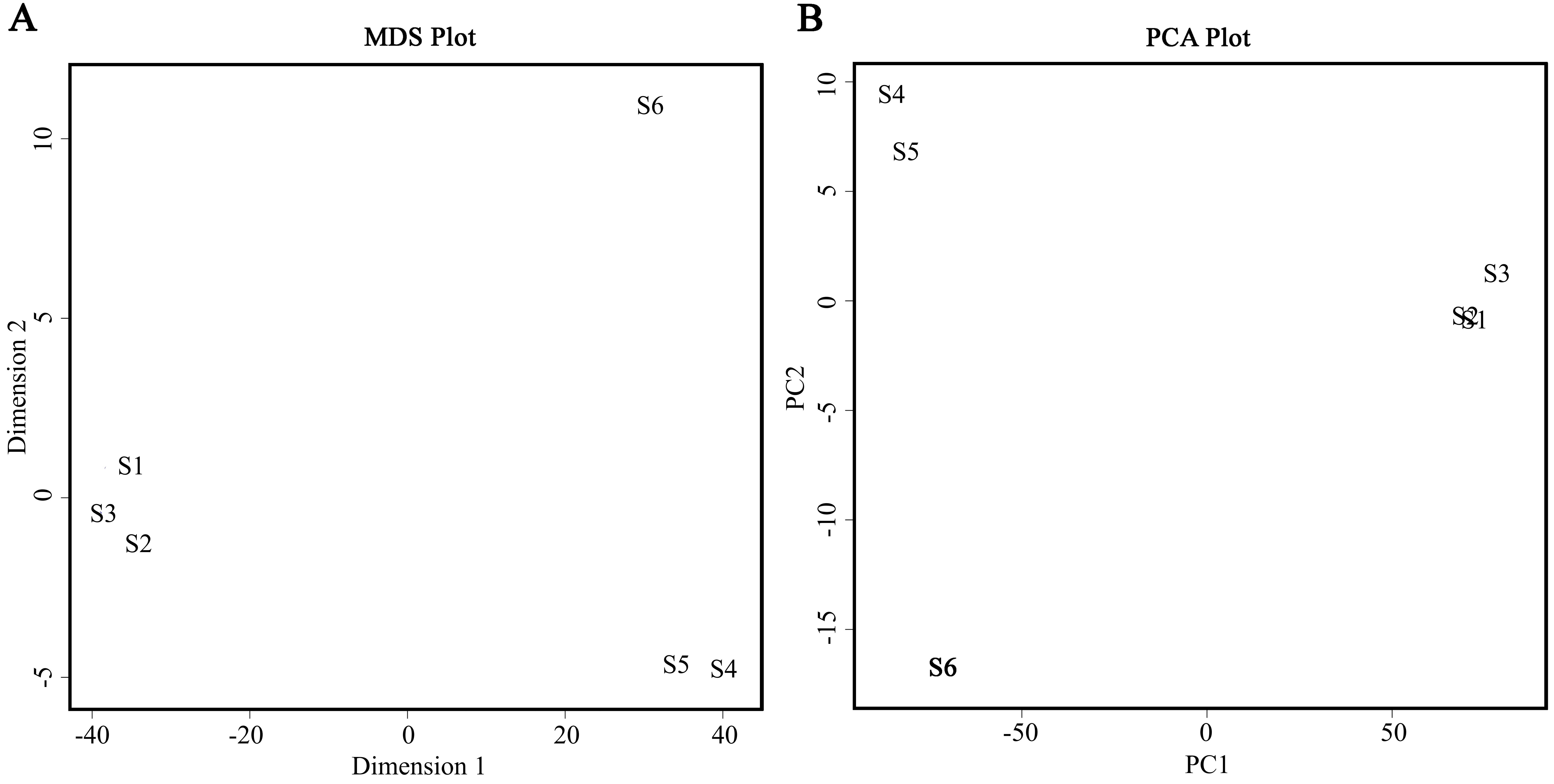

Supplementary Figure S7: Overall dispersion of data in the RNA-Seq mediated transcriptome of Arabidopsis leaves infiltrated with 1 mM CysNO. Multi-Dimensional Scatter (MDS) plot (A) and Principal Component Analysis (PCA) plot (B) showing average data dispersion of control and condition samples. These plots correspond to 3,444 up-regulated and 2,987 down-regulated DEGs shown in Figure 1A of the manuscript.
